# Natural plant-derived polysaccharides targeting macrophage polarization: a promising strategy for cancer immunotherapy

**DOI:** 10.3389/fimmu.2024.1408377

**Published:** 2024-09-16

**Authors:** Jingyang Wei, Yanpeng Dai, Ni Zhang, Zijian Wang, Xinchen Tian, Tinghao Yan, Xiaohan Jin, Shulong Jiang

**Affiliations:** ^1^ Second college of clinical medicine, Shandong University of Traditional Chinese Medicine, Jinan, China; ^2^ Institute of Chinese Medicine Processing, Shandong Academy of Chinese Medicine, Jinan, China; ^3^ Cheeloo College of Medicine, Shandong University, Jinan, Shandong, China; ^4^ College of Traditional Chinese Medicine, Shandong University of Traditional Chinese Medicine, Jinan, China; ^5^ Clinical Medical Laboratory Center, Jining No.1 People’s Hospital, Shandong First Medical University, Jining, Shandong, China; ^6^ Center for Post-Doctoral Studies, Shandong University of Traditional Chinese Medicine, Jinan, China; ^7^ Clinical Medical Laboratory Center, Jining First People’s Hospital, Jining, China

**Keywords:** polysaccharide, tumor microenvironment, macrophage, polarization, anticancer immunotherapy

## Abstract

Tumor associated macrophages (TAMs) are the predominant innate immune cells in the tumor microenvironment (TME). Cytokines induce the differentiation of macrophages into distinct types of TAMs, primarily characterized by two phenotypes: M1-polarized and M2-polarized. Cancer growth is suppressed by M1-polarized macrophages and promoted by M2-polarized macrophages. The regulation of macrophage M1 polarization has emerged as a promising strategy for cancer immunotherapy. Polysaccharides are important bioactive substances found in numerous plants, manifesting a wide range of noteworthy biological actions, such as immunomodulation, anti-tumor effects, antioxidant capabilities, and antiviral functions. In recent years, there has been a significant increase in interest regarding the immunomodulatory and anti-tumor properties of polysaccharides derived from plants. The regulatory impact of polysaccharides on the immune system is mainly associated with the natural immune response, especially with the regulation of macrophages. This review provides a thorough analysis of the regulatory effects and mechanisms of plant polysaccharides on TAMs. Additionally, an analysis of potential opportunities for clinical translation of plant polysaccharides as immune adjuvants is presented. These insights have greatly advanced the research of plant polysaccharides for immunotherapy in tumor-related applications.

## 1 Introduction

Immunotherapy has emerged as a crucial adjunctive anti-tumor modality, complementing established treatments such as surgery, chemotherapy, radiotherapy, and targeted therapies. Its significance lies in the capacity to elicit sustained remission with diminished side effects (1). Immunotherapy involves the precise identification and elimination of cancer cells by immune cells within the TME, which constitutes an intricately organized ecosystem where both cellular and cell-free components possess the capability to reprogram various facets of tumor dynamics, including initiation, growth, infiltration, metastasis, and responsiveness to anticancer therapy (2). Macrophages are acknowledged as pivotal effectors of immune responses within the TME. During the development of cancer, macrophages significantly influence the inflammatory process in the TME. Given the tumor-promoting effects of TAMs, preclinical studies on strategies to counteract TAMs have made some progress. In general, these include reducing the recruitment of TAMs and “reprogramming” TAMs (3–5). Consequently, acquiring a profound comprehension of TAMs becomes imperative to enhance the efficacy of immunotherapeutic interventions.

In the innate immune system, macrophages perform a number of critical functions, such as phagocytosis removing cellular debris, controlling infections, and maintaining dynamic tissue homeostasis. Macrophages also express different functional programs in response to different signals from the microenvironment (6). This implies that macrophages have a wide range of phenotypic states and that M1 and M2 types are the extremes of macrophage functional states (6, 7). M1-like macrophages exhibiting strong cytotoxicity and antigen-raising capacity contribute to antitumor immunity. Conversely, M2-like macrophages with immunosuppressive properties promote tumor progression (8). Circulating monocytes and tissue macrophages are co-recruited into the TME and become TAMs through various soluble or mechanical factors (9–12). TAMs are also the predominant host cells in the TME. Research evidence suggests that macrophages, an important component of TME, display tumor-fighting immune responses during initiation but shift to a protumor capacity in late-stage malignancies, supporting angiogenesis and promoting tumor migration and invasion (13). Thus, TAMs can exhibit diverse responses to TME alterations. Findings demonstrate that TAMs enrichment predicts poor prognosis and drug resistance across multiple tumor types (14, 15). Therefore, targeting macrophage polarization is a promising therapeutic strategy. Acting on the TAMs in TME to change their M2 to M1 phenotype is an intriguing and promising therapeutic approach (16, 17)..

Natural products are distinguished by their abundant origins as well as innovative and diverse structures. It has been manifested that they served as a valuable resource for the discovery of anti-tumor drugs. Natural polysaccharides derived from plants, especially plant polysaccharides used in traditional Chinese medicine, have recently attracted great interest due to their broad spectrum of bioactivities, potent therapeutic potential, and low toxicity. Extensive research indicates that plant polysaccharides exhibit biological effects such as antitumor, antioxidant, immunomodulation, regulation of intestinal microbiota, and antiviral activity (18–21). More significantly, numerous studies demonstrate that plant polysaccharides exert immune-stimulating effects on macrophages, altering their polarization state for anti-tumor phenotype. For instance, *Astragalus* polysaccharides, *Panax* polysaccharides, and *Dendrobium officinale* polysaccharides have immune-stimulating or activating effects on macrophages, primarily involving cytokine secretion, production of reactive oxygen species (ROS) and nitric oxide (NO) and the regulation of numerous signaling pathways. Thus, plant polysaccharides exhibit promising potential as immune therapy modifiers for malignancy prevention and treatment.

This review discusses the classification and sources of various natural plant polysaccharides acting on macrophages and the immunomodulatory effects of plant polysaccharides targeting macrophage polarization and provides an in-depth summary of the results of clinical translational research on plant polysaccharides as potential therapeutic agents. In conclusion, we address the difficulties and constraints associated with plant polysaccharides as possible modulators and emphasize the need for further investigations.

## 2 Macrophage polarization and immunotherapy

Macrophages, as the principal constituents of the innate immune system and consequential contributors to the adaptive immune system, manifest noteworthy efficacy in immune responses (22). The human body harbors a considerable population of macrophages, undertaking pivotal roles encompassing phagocytosis, exogenous antigen presentation, and immunoregulation through the release of cytokines and growth factors. Importantly, macrophages demonstrate substantial adaptability, marked by functional diversity. Monocytes are no longer considered merely precursor cells to macrophages. Evidence from mice and humans that tissue macrophages originate from embryonic and adult circulating myeloid precursors (10). In many mouse tumor models, circulating monocytes are the main precursors of TAMs (13, 23). In the context of human bone marrow transplantation, lymphoma-associated macrophages were found to originate from myeloid precursors (24).

**Figure 1:**
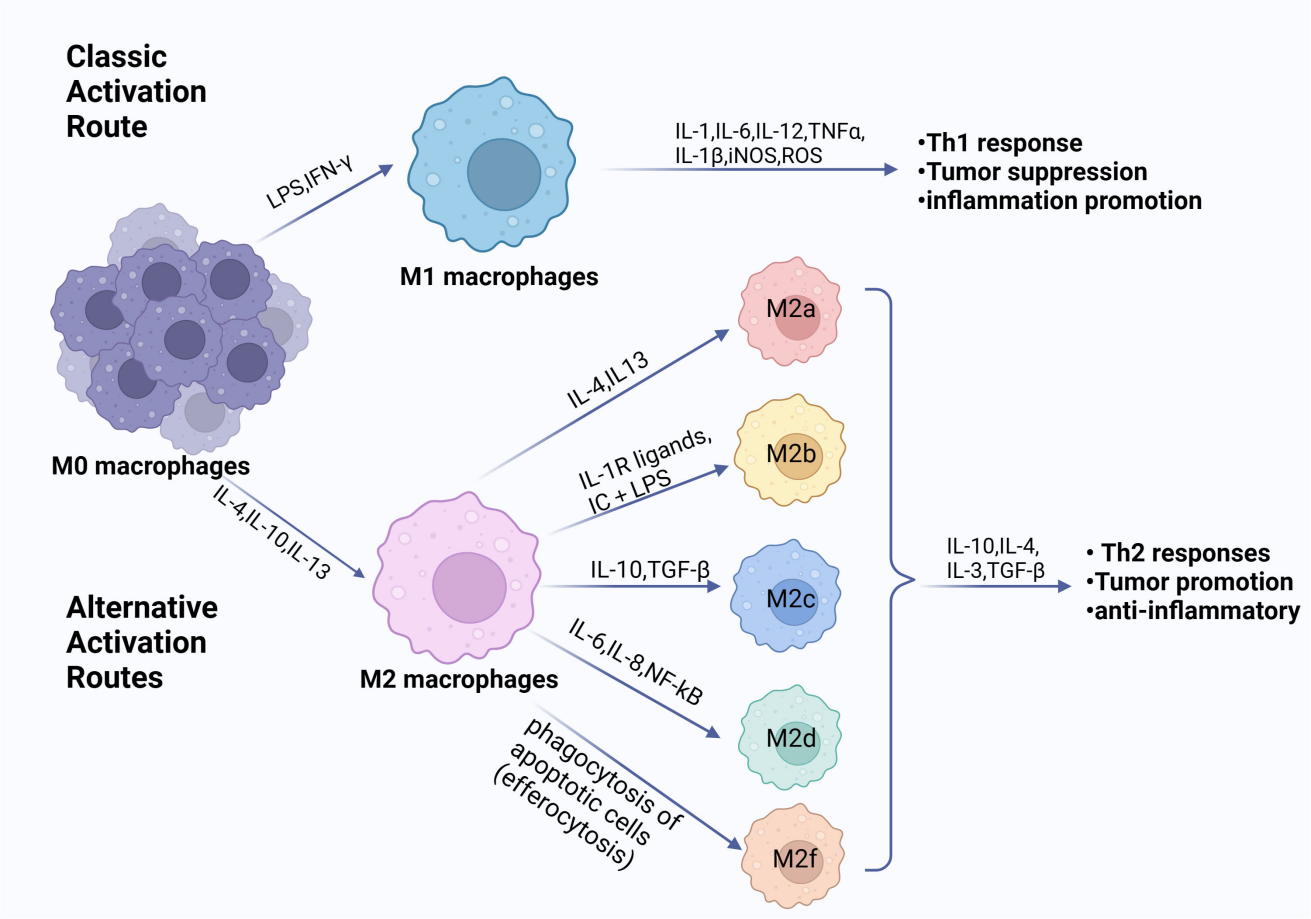
Phenotypes of macrophage polarization. Exposure to diverse cytokine environments induce monocytes’ differentiation towards polarized macrophage subpopulations. When exposed to LPS, IFN-γ or other microbial products, monocytes differentiate into M1 macrophages. When exposed to IL-4, IL-10, IL-13, and immunosuppressive agents, monocytes differentiate into M2 macrophages. The M1 and M2 subpopulations are functionally and phenotypically distinct. The M1 cells exert an antitumorigenic effect. Conversely, the M2 cells contribute to a pro-tumorigenic milieu. (Created with BioRender.com).

When exposed to various stimulus signals, macrophages enter a condition known as “macrophage polarization,” which changes their morphology, function, and phenotype (25, 26). The classical concept divides polarized macrophages into two categories: M1 classical activated macrophages and M2 alternative activated macrophages. The two polarization states are shown in **Figure 1**. Depending on the type of inducer and expression marker, M2 macrophages can be categorized into a number of different subtypes, including M2a, M2b, M2c, M2d, and M2f (27). However, the expression of all subtypes *in vivo* remains unknown (28). M1 macrophages are activated by lipopolysaccharide (LPS) and cytokines (predominantly IFN-γ and IL-2) exhibiting high levels of Toll-like receptors 2 and 4, CD80, CD86, and MHC class II (26). They are able to produce large amounts of inflammatory factors (IL-1β, IL-6, and TNF-α, etc.) and release NO and ROS, which play an important role in pathophysiological processes such as killing pathogens, resisting parasites and tumor cells, and pro-inflammatory responses (22, 25). M2 macrophages, induced by IL-4, IL-33, and TGF-β stimulation, usually expressing CD206 and CD163, are regulated by a variety of transcription factors and secreted cytokines in regulating tumor growth, thereby modulating inflammation, suppressing immune response, and stimulating cellular and tissue remodeling, angiogenesis and tumor progression (29, 30).

Additionally, macrophages demonstrate adaptability by modulating the TME as a tumor advances. It is noteworthy that not all TAMs manifest the M2 phenotype. Intriguingly, TAMs undergo a phenotypic transformation to M2 in hypoxic TME conditions, thereby promoting tumor progression through the secretion of immunosuppressive cytokines and consequent inhibition of immune effector cells (6, 25, 31). In addition to cytokine secretion, there are several immunosuppressive receptors on the surface of macrophages, such as sialic acid-binding immunoglobulin-type lectins (Siglecs), signal-regulating protein alpha (SIRPα), leukocyte immunoglobulin-like receptor B (LILRB), macrophage receptor with collagen structure (MARCO), and Clever-1 (32–36). Cancer cells express anti-phagocytic surface proteins CD24 and CD47 that interact with Siglec-10 and SIRPα, respectively, triggering “don’t eat me” signals to evade immune surveillance and immune clearance (37, 38). Shen et al. used CD24/Siglec-10 blocking peptide (CSBP), which blocks the interaction between CD24/Siglec-10 and PD-1/PD-L1, to enhance macrophage-mediated phagocytosis of tumor cells and activate CD8 T cells (39). The molecule Clever-1 is expressed in M2-polarized macrophages. Targeting Clever-1 is anticipated to enhance existing immunotherapy approaches by enabling T-cell and macrophage-mediated anticancer immunity (36). We discuss current strategies for targeting macrophages, which include (1) altering the composition of TAM cells (2); reprogramming TAM cells to polarize M2 to M1 (3); modulation of macrophages by cytokines; and (4) functional blockade of immunosuppressive macrophages, such as Siglec-9/10, SIRPα, MARCO, LILRB2, and Clever-1. Macrophage-based immunotherapies are expected to advance immuno-oncology in the coming years.

## 3 Natural plant polysaccharides as modulators of macrophage polarization

Plant polysaccharides are polymers consisting of multiple monosaccharides linked by glycosidic bonds, produced by plant cell metabolism. Current research on plant polysaccharides focuses on extraction and purification, structural characterization and analysis of immunomodulatory activities (40–44). The majority of plant polysaccharides predominantly interact with both the innate and adaptive immune systems, thereby augmenting host immunity and indirectly exerting suppressive effects on tumors (21, 45, 46). Especially, plant polysaccharides have significant effects on the regulation of immune responses by altering the activity and activities of macrophages. This, in turn, contributes to their anti-tumor and immune regulatory properties. They play a role in controlling the activity of macrophages and adjusting the levels of inflammatory cytokines, such as TNF-α and IL-1β, in order to coordinate a suitable inflammatory reaction. Moreover, these polysaccharides have the potential to improve the process of macrophage phagocytosis, therefore facilitating the elimination of pathogens or aberrant cells. Furthermore, it is believed that they regulate the polarization state of macrophages, influencing the intricate equilibrium between their M1 (pro-inflammatory) and M2 (anti-inflammatory) states. Plant polysaccharides therefore show great promise as bioactive modulators in tumor therapy and open up new options for the synthesis of novel immunomodulatory medications.

### 3.1 Classification, sources of natural plant polysaccharides acting on macrophages

It has been noted above that polysaccharides with the potential to modulate macrophage function have been found in a variety of plants. The fractions and biological activities of certain plant polysaccharides are listed in **Table 1**. 

**Table 1:** Immunomodulatory activity of natural plant polysaccharides on macrophages.

Botany	Polysaccharides	Monosaccharide composition	Models	Effects on macrophages	Ref.
Rosa setate x Rosa rugosa waste	WSRP-2a	GalA, Ara, Gal, Rha, and Man	RAW264.7	Promote proliferation, NO release, and the secretion of TNF-α and IL-6	(47)
	WSRP-2b	GalA, Ara, Gal, Rha, Man, Glc, Xyl, and GlcA			
Astragalus polysaccharide	APS	Glc, Gal, Rha, Ara, Fru, Man, and GalA	RAW264.7	Stimulate macrophages to secrete NO and TNF-α, IL-2, and IFN-γ	(48)
maca (Lepidium meyenii Walp.)	LMP-1	Glc and Ara	RAW264.7	Activate TLRs/NF-κB signaling pathway; stimulate TNF-α, IL-1b and IL-6	(40)
Asparagus officinalis L.	WASP	Rha, Ara, Gal, Glc, Xyl, and Man	RAW 264.7	Increase the release of IL-6, TNF-α, and IL-10 and improve the expression of mRNA	(49)
Hovenia dulcis peduncles	HDP3A	GalA, Gal, Rha, Ara, Xyl, Fuc, Man, and Glc	RAW 264.7	Stimulate the proliferation of RAW264.7 cells	(50)
Allium sativum L.	GPSs	Fuc, Rha, Gal, Glc, and Fru	RAW264.7	Stimulate NO	(51)
Angelica sinensis (Oliv.) Diels	APS-3a	Glc, Gal, Ara, Rha, and Man	Male BALB/c mice peritoneal macrophage		(52)
	APS-3b	Glc, Gal, Ara, Rha, and Man		Enhance the peritoneal macrophages phagocytosis; increase the release of TNF-α, NO	
	APS-3c	Glc, Gal, Ara, Rha, Man, and Xyl		Increase the release of TNF-α, NO	
Lepidium meyenii (maca)	MC-1	Ara, Man, Glc, and Gal	RAW 264.7	Enhance the pinocytic and phagocytic capacity; promote the NO, TNF-α and IL-6 secretion	(53)
	MC-2	Ara, Man, Glc, and Gal	RAW 264.7	Induce M1 polarization of original macrophages and convert M2 macrophages into M1 phenotype	(54)
Aloe vera L. var. chinensis (Haw.) Berg.	PAC	Man, Gal, Glc, and Ara	BALB/c mouse peritoneal macrophages	Stimulate TNF-α, IL-1b; stimulate peritoneal macrophage proliferation	(55)
Citrus grandis	HPP-1	Rha, Ara, Fuc, Man, and Gal	RAW264.7	Stimulate NO, TNF-α, and IL-6 secretions; activate NF-κB and MAPK signaling pathways	(56)
Nelumbo nucifera Gaertn.	LLWP-C	Rha, Ara, Gal, Glc, and GalA	RAW264.7	Stimulate NO, TNF-α, IL-1β, IL-6, and IL-12; activate MAPK and NF-ĸB signaling pathways	(57)
Stem lettuce	SLP	Man, Rha, GalA, Gal, and Ara	RAW264.7	Promot proliferation, phagocytosis and NO production	(58)
Rosa laevigata Michx	PPRLMF-2	Rha, Ara, Xyl, Man, Glc, Gal, and GalA	RAW264.7	Induce NO, INF-α, and IL-6; activate MAPKs and NF-κB signaling pathways	(59)
black radish (Raphanus sativus ver niger)	BRHE	Glc, Rha, Fuc, Xyl, GalA, Ara, and Gal	RAW264.7	Stimulate NO, ROS, IL-1β, IL-6, and TNF-α; stimulate iNOS and COX-2 proteins; induce TLR2/4–MAPK–NFκB–Akt–STAT3 signaling pathway; induce the promotion of macrophage phagocytosis	(60)
Gardenia jasminoides Ellis	GP2a	GalA, Ara, Gal, Glc, Rha, Man, GlcA, Xyl, and Fuc	RAW264.7	Stimulate NO, TNF-α, IFN-γ, IL-1β, IL-6, and GM-CSF	(61)
Abrus cantoniensis	ACP	Glc, Rha, Gal, GalA, GlcA, and Man	RAW264.7	Stimulate ROS, NO, iNOS, TNF-α, IL-6, and IL-1b; induce MyD88/Akt/MAPKs signaling pathway; enhance the pinocytic and phagocytic capacity	(62)
Raspberry Pulp	RPP-2a	Rha, Ara, Gal, Glc, Xyl, GalA, and GlcA	RAW264.7	Stimulate NO, TNF-α, IL-6, IL-1β, and iNOS	(63)
Lycium barbarum (L. barbarum)	LBP	Gal, Glc, Rha, Ara, Man, and Xyl	BALB/c mice peritoneal macrophages	Stimulate CD40, CD80, CD86 and MHC class II; enhance endocytosis and phagocytosis	(64)
			RAW264.7	Activate AP-1 and NF-κB; induce TNF-α, IL-1-β, and IL-12p40 mRNA expression;	
raspberry (Rubus idaeus L.)	RPP-3a	Rha, Ara, Gal, Glc, Man, and GalA	RAW264.7 murine macrophage cell	Stimulate NO, TNF-α, IL-6, iNOS, and IL-1β	(65)
Radix Aconiti Lateralis Preparata (Fuzi)	FZPS -1	D-Ara and D-Glc	RAW264.7	Promote macrophage phagocytosis; stimulate NO, IL-6, IL-1, and TNF-α	(66)
Achyranthes bidentata Blume	ABPS	Fru, Glc	J774 A.1 cell line (mouse monocyte/macrophage)	Stimulate IL-1β and TNF-α; induce TLR4/MyD88/NF-κB signaling pathway	(67)
Cyclocarya paliurus	S-CP1-8	Ara, Rha, Gal, Glc, Xyl, Man, GalA, and GlcA	RAW264.7	Stimulate NO, TNF-α, IL-1β, and IL-6	(68)
Lilium lancifolium Thunb.	LLP-1A	Man and Glc	RAW264.7	Stimulate NO, IL-6, TNF-α, and IL-1β; induce TLR4-mediated NF-κB signal pathway	(69)
Carthamus tinctorius L.	SF1, SF2	GlcA, GalA, Glc, and Ara	Female C3H/HeN (5to 6week old) mice	Stimulate IL-1, IL-6, IL-12, IFN-γ, and TLR4	(70)
Schisandra chinensis (Turcz.) Baill	SCPP11	Rha, Man, Glc, Ara, and GalA	ICR mice	Increase pinocytic activity; increase immunoglobulin levels, cytokines levels	(71)
			RAW264.7	Stimulate iNOS and TNF-α mRNA	
Glycyrrhiza uralensis fish	GP	Gal, Glc	Male BALB/c mice peritoneal macrophages	Stimulate NO, IL-6, and IL-12	(72)
Platycodon grandiflorum	PG	Fru	BDF1 mice peritoneal macrophages	Stimulate NO	(73)
Astragalus membranaceus (Fisch) Bge.; Huangqi	RAP	Rha, Ara, Glc, Gal, and GalA	RAW264.7	Stimulate NO, TNF-α, IL-6, and iNOS	(74)
Polygonatum sibiricum	PSP	Rha, Ara, Xyl, Man, Glc, and Gal	RAW264.7	Stimulate NO, IL-1β, IL-6, IL-12p70 and TNF-α; activate TLR4-MAPK/NF-κB signaling pathways	(75)
Apple	AP	Man, Rha, GalA, GalA Glc, Gal, Xyl, Ara, and Fuc	RAW264.7 murine macrophage cell	Upregulate the TLR4/NF-κB signaling pathway; switch M2 macrophages to M1 phenotype	(76)
Codonopsis pilosula endophyte	DSPS	Gal, Glc, Rha, Fuc, Ara, and Man	RAW264.7	Promote macrophage polarization toward M1 phenotype;	(77)
Ilex asprella	IAPS-2	Gal, Glc, Rha, and Ara	RAW264.7	Enhance M1 type differentiation in TAMs	(78)
			C57BL/6J mice, female	Stimulate IL-12, NO, MHC II, and INF-γ	
Cyclocarya paliurus	CPP-3	Rha, Ara, Xyl, Man, Glc, and Gal	RAW264.7	Increase the amount of NO, TNF-α, IL-1β, and PGE2 released	(79)
Smilax glabra Roxb	SGRP1	Man, Fuc, and Glc	RAW264.7	Promote the phagocytosis and increase macrophage-derived biological factors including NO, IL-6, TNF-α and IL-1β secretion	(80)
Asparagus cochinchinensis	ACMP	Man, Rha, GalA, and Xyl	RAW264.7 cells and BMDM cells	Regulate immunological function through the TLR4-MAPK-JNK/ERK/p38 signaling pathway	(81)

The biological activity of polysaccharides is related to their chemical composition and structure, such as molecular weight (Mw), conformation, and glycosidic bonding (89). There are large differences in the antitumor activity of polysaccharides composed of different monosaccharides. The majority of plant polysaccharides based on glucose (Glc) and rhamnose (Rha) currently exhibit strong anti-tumor action; the more Glc there is in the polysaccharide, the more anti-tumor activity there is (49–51). While some polysaccharides have only one monosaccharide component, others are made up of complicated sets of monosaccharides. In contrast to the polysaccharides isolated from *Smilax glabra Roxb*, which consisted of mannose (Man), fucose (Fuc), and Glc, all three polysaccharides derived from *Cistanche deserticola* were determined to be composed of Glc (80, 90). Furthermore, various fractions of plant polysaccharides can be isolated from a single plant, and each polysaccharide displays distinct functional effects. For example, WSRP-2a and WSRP-2b, both pectic polysaccharides, were isolated from *Rosa setate* x *Rosa rugosa* waste (47). These two fractions were mainly composed of glucuronic acid (GlcA), galacturonic acid (GalA), arabinose (Ara), galactose (Gal) and Rha, but the average molecular weights varied considerably, 56.8 and 23.9 kDa, respectively (47). WSRP-2b exhibited higher α-amylase and α-glucosidase inhibitory activities, which may be related to the higher content of glucuronides or lower relative molecular mass of WSRP-2b (91). The effect of WSRP-2a on the RAW264.7 cell proliferation and cytokine (TNF-α and IL-6) secretion with strong stimulatory effect and more immune-enhancing activity (47). The conformational relationship of pectic polysaccharides is not clear, and Wu et al. hypothesized that the different bioactivities may be due to different molecular weights (47).

Polysaccharides derived from edible or medicinal plants have several effects on macrophages, including increasing their phagocytic activity, inducing the expression of various cytokines and chemokines, upregulating ROS and NO production, and inducing either the M0 to M1 transition or the polarization of M2 to M1 states. For example, *Astragalus polysaccharide* (PG2), a principal active constituent from *Astragalus membranaceus* root, displays robust bioactivity *in vitro* and *in vivo* studies, being efficiently employed for use in the treatment of cancer and other diseases (92). Bamodu et al. demonstrated by *in vitro* and *in vivo* experiments that PG2 dose-dependently and significantly increased the polarization ratio of M1 macrophages and down-regulated IL-4- and IL-13-induced M2 polarization in non-small cell lung cancer (NSCLC) (93). RAP is a purified polysaccharide extracted from *Radix Astragali* polysaccharides containing Rha, Ara, Glc and Gal, with a backbone consisting of 1,2,4-linked Rhap, α-1,4-linked Glcp, α-1,4-linked GalAp6Me and β-1,3,6-linked GalP (94). Wei et al. demonstrated that RAP induced the expression of M1 marker genes such as iNOS, IL-6, TNF-α, and CXCL10, attenuated 4T1 cell growth, and transitioned macrophages towards an M1 phenotype or reversed M2 polarization to M1 (74).

To demonstrate the targeting of plant polysaccharides on macrophages, clodronate liposomes are a well-established method of depleting macrophages (95). Wang et al. depleted and replenished macrophages within C57BL/6 mice to further demonstrate that *Dendrobium officinale* polysaccharides can inhibit tumor growth by promoting polarization of M1 macrophages (96). In addition, studies on the mechanisms reveal that the TLRs- NF-κB pathway and the activated AMPK- PPARs pathway contribute to the anti-tumor effect of polysaccharides *in vitro* and *in vivo*. Apple polysaccharides (AP) have a relative molecular mass of 5,000-10,000 Da and their main components are GalA and Gal (76). Sun et al. found that AP not only increased macrophage M1 markers (iNOS, TNF -α, IL -23) and decreased macrophage M2 markers (TGF-β, IL -4, IL -10), but also converted M2 macrophages to M1 phenotype via TLR-4 signaling (76).

### 3.2 Mechanism of plant polysaccharides activating macrophages

Plant polysaccharides regulate immunity in a multifaceted modulatory manner, with a clearer mechanism observed in macrophages. Specifically, plant polysaccharides stimulate the release of cytokines such as TNF-α, IL-6, and NO, thereby promoting macrophage differentiation toward the M1 phenotype (76, 93). Simultaneously, research has elucidated the molecular mechanism of polysaccharide immunomodulation. Plant polysaccharides interact primarily with macrophage surface receptors, encompassing the mannose receptor (MR), Toll-like receptors (TLR2 and TLR4), and Dectin-1 receptor, or other derivatives (41). Macrophages are activated and stimulate signal transduction pathways leading to transcriptional activation and production of inflammatory factors.

#### 3.2.1 Regulation of cytokines and chemokines

**Figure 2:**
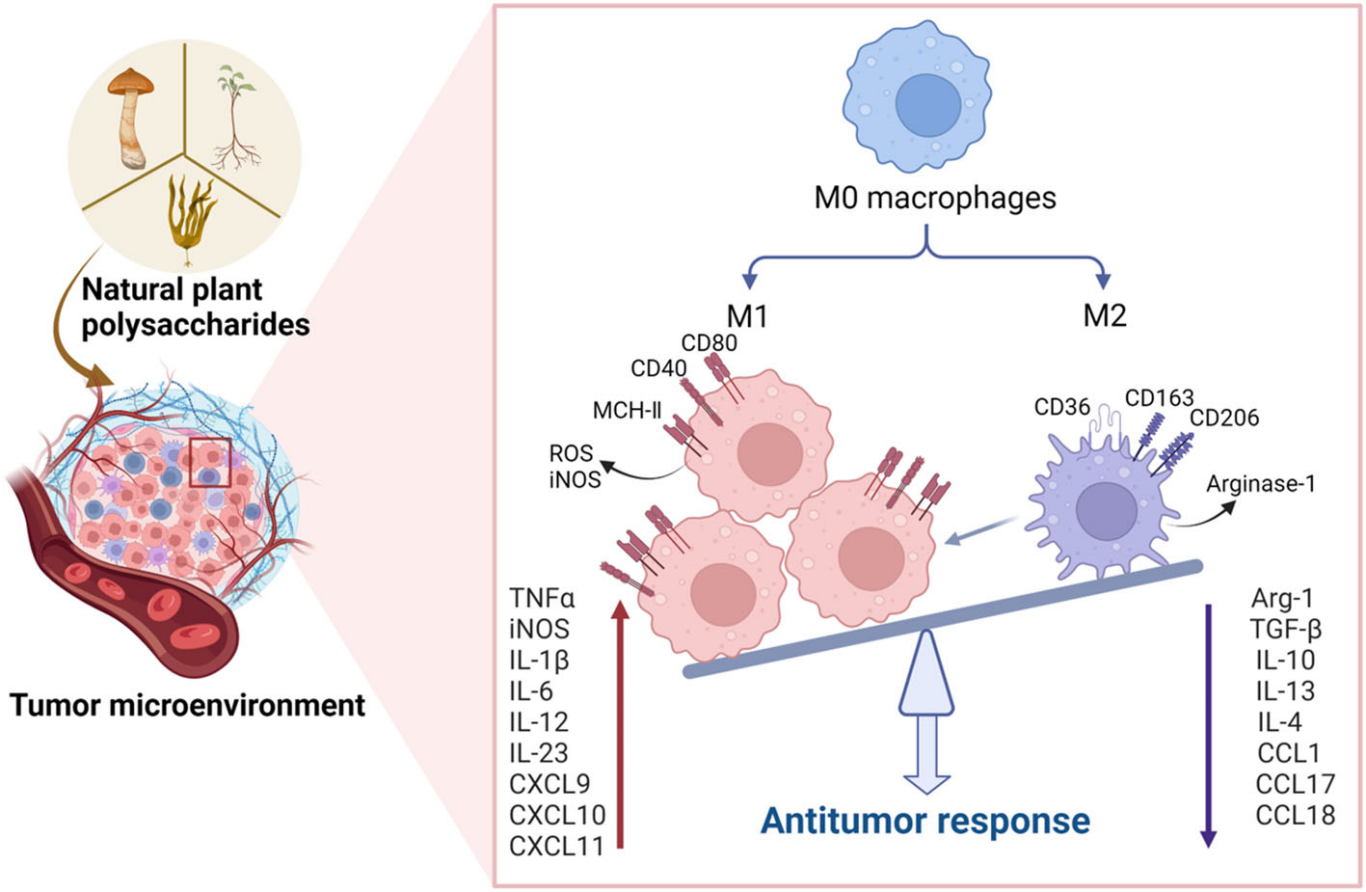
Natural plant polysaccharides act to polarize the M2 phenotype to the M1 phenotype in the TME. In addition to directly inducing apoptosis in tumor cells, polysaccharides exhibit the capacity to impede tumorigenesis and progression by influencing the TME. Specifically, these natural polysaccharides enhance the expression of M1 cytokines, including IL-6, IL-12, TNF-α, and IL-23, while concurrently inhibiting the expression of M2 cytokines such as IL-10, IL-13, TGF-β, and IL-4 within the TME. This dual action underscores the potential therapeutic efficacy of natural polysaccharides in the intricate regulation of TME, thereby presenting a promising avenue for cancer treatment strategies. (Created with BioRender.com).

Cytokines serve as crucial mediators in orchestrating the interplay between immune and non-immune cells within the TME (97). Notably, cytokines like IL-2, IL-6, TNF-α, and IFN-γ, known for their inflammatory enhancement properties, contribute to stimulating tumor cell immunity, thereby fostering anti-tumor activity (60). Conversely, cytokines such as IL-10, IL-13, and TGF-β operate by inhibiting inflammation and suppressing immune cells, consequently creating an environment conducive to tumor progression (15). **Figure 2** demonstrates that natural plant polysaccharides modulate the production and secretion of cytokines involved in polarization.

Three acidic polysaccharides (APS-3a, APS-3b, and APS-3c) were extracted from *Angelica sinensis* (Oliv.) Diel by Cao et al. Among them, APS-3b and APS-3c, but not APS-3a, showed significant antitumor effects *in vivo* (52). The reason for the different anti-tumor activity functions may be related to the chemical structure (e.g., relative molecular mass, monosaccharide composition) of these acidic polysaccharides. Compared to APS-3a (5.9×105 Da), APS-3b and APS-3c had lower molecular weights (2.3×105 Da and 1.4×104 Da) (52). APS-3a and APS-3b have the same monosaccharide composition, while APS-3c contains more xylose (Xyl) (52). Each polysaccharide also contains different major monosaccharides. Glc is the primary monosaccharide of APS-3a, Ara is the main monosaccharide of APS-3b, and Man, Rha, and Glc are the major monosaccharides of APS-3c (52). In order to clarify the connection between the architectures of the three acidic polysaccharides and their functional activities, more research is required. Im et al. purified the polysaccharide SHP in *Salicornia herbacea* and found that the combination of SHP and IFN-γ synergistically inhibited the growth of mouse RAW 264.7 and stimulated the secretion of cytokines such as TNF-α and IL-1β from RAW264.7 (98). Zhang et al. identified, MC-2, a heteropolysaccharide consisting of Ara, Man, Glc and Gal extracted from Lepidium meyenii (maca) (54). MC-2 increased the concentrations of IL-6 and iNOs, whereas the levels of IL-10 and arginase-1 (Arg-1) remained unchanged, suggesting that MC-2 induces macrophage polarization toward the M1 phenotype. However, the effect of MC-2 on macrophage polarization is limited. In addition, They found that MC-2 markedly enhances IL-6 and iNOS mRNA production in IL-4-induced M2 macrophages, suggesting that MC-2 can convert M2 macrophages into M1 (54). PG2 dose-dependently enhanced M1 polarization while downregulating IL-4 or IL-13-induced M2 polarization. High M2/M1 status in TME is often associated with poor prognosis in most solid tumors (99). Consequently, PG2-induced M2 macrophage elimination offers an innovative approach to immune therapy in non-small cell lung cancer patients (93).

Chemokines regulate macrophage polarization. Studies have shown that CCL19, CCL21, CCL24, CCL25, and CXCL10 specifically induce M1 macrophage chemotaxis (100). TAMs secrete CCL3 (101), CCL5 (102), CCL15 (103), CCL18 (104), and other chemokines that can promote tumor metastasis, contribute to angiogenesis, and enhance immunosuppression and cancer cell resistance post-chemotherapy. Liu et al. concluded that macrophage-secreted CCL5 stabilizes PD-L1 *in vitro* and *in vivo*, suppressing T-cell killing of HT29 cells, and thereby promoting immune escape (105). Therefore, comprehending the function of chemokines within TME and manipulating them therapeutically offers potential strategies for cancer treatment (106).

#### 3.2.2 NO and ROS generation

NO mediates cell death, eliminates infectious organisms, and functions as a signaling molecule (107). A growing number of studies reveal that iNOS mediates NO upregulation post-LPS macrophage activation, leading to mitochondrial dysfunction and tricarboxylic acid cycle disorder, resulting in macrophage transformation into M1 (108). Thus, NO has become an important marker for the transformation of M2 macrophages into M1 macrophages and enhanced tumor suppressor conditions (109). Zhou et al. reported that APS were able to directly increase NO production by macrophages *in vitro*, participate in pathogen clearance, and promote tumor cell destruction by activated macrophages (110). *Lily* polysaccharides can enhance immune function by significantly inducing NO production in macrophages in a dose-dependent manner (69). The structure of water-soluble polysaccharides extracted from *juniper cones* contains type II arabinogalactans, which were analyzed by Schepetkin et al. for their ability to induce iNOS and NO production in macrophages (111).

ROS is essential for the induction and maintenance of M1-type macrophage polarization. It has been reported that ROS promotes the expression of pro-inflammatory genes in macrophages and interferes with macrophage differentiation by stimulating the NF-κB and P38MAPK signaling pathways. BRHE, an extract isolated from black radish, was able to induce ROS production in RAW264.7 cells, and ROS are involved in immunostimulatory functions through phagocytic activation (60). The innate immune response is aided by phagocytosis, the initial reaction of an activated macrophage to invasive pathogens or microbes. Activated macrophages secrete more cytokines such as IL-6 and TNF-α, which act on pathogens and cancer cells (112). Thus, reducing the growth advantage of tumor cells is possible through balancing ROS generation and antioxidant defense (113).

#### 3.2.3 Regulation of surface receptor expression

Plant polysaccharides primarily activate macrophages through the recognition of polysaccharide polymers by certain receptors. These receptors include TLRs, mannose receptors (MR), Dectin-1 receptors, complement receptors (CRs), scavenger receptors (SR), and others. Numerous studies have shown that TLRs play an essential role in the macrophage response to many microbial infections. Polysaccharides interacting with TLRs mainly contain glycosidic bonds of the α-(1→3), α-(1→4), β-(1→3), and β-(1→4) types (114, 115). One such receptor, TLR4, is necessary for many polysaccharide-recognition signaling events (116). In response to pathogen invasion, inflammatory cytokines such as IL-17, TNF, IFN-γ, IL-6, and IL-2 are produced when TLR4/TRAF6/NF-κB signaling is triggered (117). For example, MC-2 polysaccharides exhibit elevated glucose levels, particularly β- (1, 3)-Glc, β- (1, 4)-Glc, and α-(1→4)-Glc, which are consistently associated with TLR4 (54). In addition, TLR4 receptors-mediated signaling pathway is a common pathway for cytokine release in *Lepidium meyenii* (118), *Panax* (25), *Lycium barbarum* (119), and *Achyranthes bidentata* (67).

A crucial part of the early immune response, MR is a member of the C-type lectin receptor family and is expressed on the surface of macrophages. Due to the effect of ligands and co-receptors, MR is extensively implicated in a range of inflammatory reactions (120). The target receptor for *Aloe vera* polysaccharides may be the MR receptor of macrophages, which may bind to the MR of macrophages and lead to immune activation (55).

As pattern recognition receptors, SR work in tandem with other PRRs to identify and eradicate microorganisms in reaction to the production of cytokines. It has been shown that binding of SR and CR3 to their ligands activates phospholipase C (PLC), and the products of PLC cleavage activate protein kinase C (PKC) and phosphatidylinositol 3-kinase (PI3K), leading to activation of mitogen-activated protein kinases (MAPK), extracellular signal-regulated kinase (ERK), and NF-κB, which ultimately triggers gene transcription events (121). MARCO is a member of the class A scavenger receptor (SR-A) family, which is widely expressed in TAMs (35). The findings suggest that MARCO(+) TAMs is negatively associated with prognosis in some liver, lung and breast cancer cases (122–124). Eisinger et al. applied MARCO-targeting antibodies, which changed inhibitory TAM into pro-inflammatory TAMs (125). On the other hand, SR-mediated plant polysaccharides with various conformations, including α and β conformations, increase phagocytosis by macrophages and induce dendritic cell maturation. If we can find the targeting relationship between plant polysaccharides and MARCO receptors in TAMs, it provides new ideas for macrophage immunotherapy.

The primary β2 integrin that is known to aid in innate immune cells’ detection of fungi is called CR3. The two ligand binding sites on CR3, the I domain and the lectin-like domain, bind to β-glucan and protein ligands, respectively (126). Most polysaccharides coupled to CR3 receptors have a β-configuration in their shape, thus stimulating polysaccharides improve phagocytosis of phagocytes, boost cytokine release, and fortify the immune system (127). Expression of CD14 in macrophages leads to pro- or anti-inflammatory responses (128). CD14 was also shown to be involved in the response to plant polysaccharides. Han et al. isolated a fructan from the radix of *Platycodon grandiflorum* and demonstrated that pretreating peritoneal macrophages with anti-CD14 or CD11b antibodies significantly reduced macrophage NO induced by tangerine polysaccharides, indicating that these surface molecules may be potential targets for polysaccharides (73). Dectin-1 is another pattern recognition receptor (PRR) that can be seen in macrophages and dendritic cells. Studies have reported that activation of Dectin-1 leads to cytokine release and ROS generation (129). In addition, Dectin-1, together with TLR2 and TLR4, can synergize to promote TNF-α production by human macrophages (130).

#### 3.2.4 Signaling pathways

**Figure 3:**
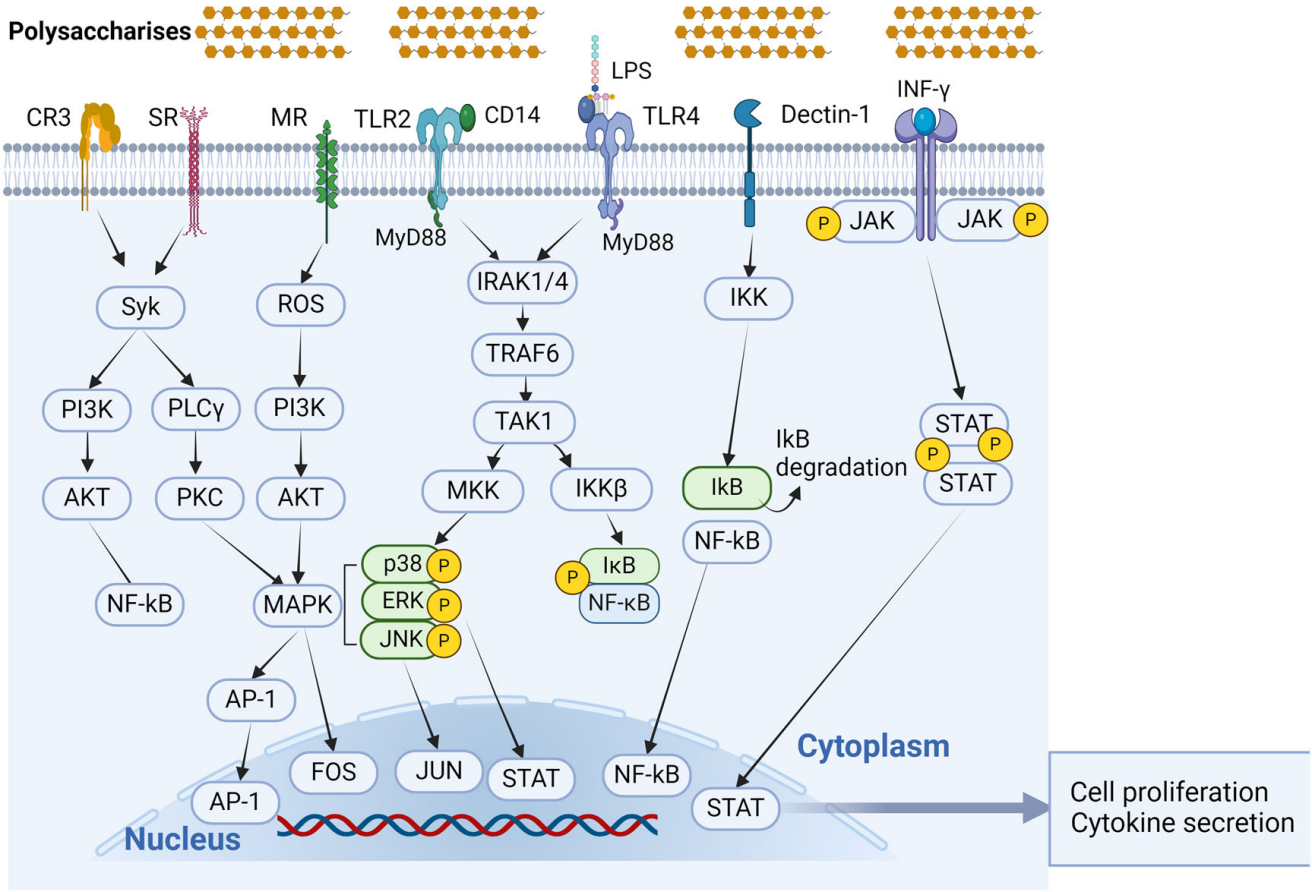
Signal transduction pathways associated with polysaccharide immunomodulation in macrophage activation. Phytopolysaccharides can activate macrophages through different receptor kinks, such as TLR4, TLR2, CR3, MR, SR, and Dectin-1. All of these receptors can function independently, and in certain cases, they may combine together to form complexes in signaling. (Created with BioRender.com).

With the in-depth study of the immunomodulatory mechanisms of plant polysaccharides, attention has shifted from the extracellular to the intracellular level in the search for new targets (131). Once activated macrophage receptors can initiate a series of signaling pathways that lead to activation of transcription and production of associated cytokines that promote macrophage polarization (55, 78, 93, 132). Macrophage differentiation is influenced by a number of variables, including some microbial products and inflammatory cytokines. Factors that stimulate M1-type macrophages include NF-κB, MAPKs, activator protein 1 (AP-1), signal transducer and activator of transcription 1 (STAT1), interferon regulatory factor (IRF) 5, and serine/threonine kinase (AKT) 2, whereas factors that stimulate M2-type macrophages include STAT6, IRF4, peroxisome proliferator activated receptor (PPAR) γ, and AKT1 (20). **Figure 3** shows the action pathway of plant polysaccharides.

##### 3.2.4.1 Toll-like receptor signaling pathway

Macrophages rely significantly on TLRs as PRRs to initiate immune responses. Notably, TLR2 and TLR4 play pivotal roles in recognizing signals associated with polysaccharides, effectively transmitting them to intracellular signaling pathways (133). Many studies have shown that plant polysaccharides can bind to TLR2 and TLR4, activate downstream signaling pathways, and exert immunomodulatory effects (69). However, TLR2 and TLR4 have different affinities for polysaccharides. Jeon et al. reported that radish polysaccharides-mediated immunomodulatory activity in RAW264.7 cells requires two major receptors, TLR2 and TLR4. The immunological response can be facilitated by both TLR4 and TLR2 signaling, which are both activated by radish polysaccharides signaling; however, the affinity of TLR4 for radish polysaccharides is much higher than that of TLR2 (60). The experiment conducted by Qu et al. demonstrated that *Abrus cantoniensis* polysaccharides (ACP) had a greater impact on TLR4 expression than TLR2, suggesting that TLR4 is the major pattern recognition receptor for ACP in macrophages (62). TLR4 expressed by macrophages is essentially involved in many natural plant polysaccharide-induced events. TLR4 signaling can be regulated through MyD88-dependent or MyD88-independent pathways (134). Myeloid differentiation factor 88 (MyD88), a key downstream signaling ligand in the TLR4 signaling pathway, drives NF-κB into the nucleus, activates related genes transcription, enhances inducible nitric oxide synthase, NO, and cytokines, and activates T cells for immune responses (135). The polysaccharide extracted from the dried rhizomes of *Atractylodes macrocephala* Koidz is a homogeneous polysaccharide composed of Glc, which is mainly connected by β-D-1→3 and β-D-1→3.6 It has a simple structure and small molecular weight. Liu et al. found that it stimulated the immune-regulatory function of the TLR4-MyD88-NF-κB signaling pathway (136). Similarly, *Achyranthes bidentata* polysaccharide, a dried root extract of *Achyranthes bidentata* Blume, as a fructan, activates TLR4 signaling through the MyD88-dependent pathway (67).

##### 3.2.4.2 MAPK signaling pathway

The MAPK family includes three key kinases: p38, JNK, and ERK. These kinases are involved in cell proliferation, migration, invasion, and angiogenesis, and are important for cell development. Phosphorylation of particular substrates is carried out by each subclass through its own distinct activation pathway (137). The primary role of p38 is to cause cell apoptosis and initiate the synthesis of pro-inflammatory substances such as TNF-α and COX-2 (138). ERK is mainly involved in macrophage growth and differentiation (139). Multiple intracellular signaling pathways induced by plant polysaccharides ultimately converge on the MAPK pathway, which regulates macrophage NO and cytokine production and secretion (140). Examples include Black Radish polysaccharides (40), *Lycium barbarum* polysaccharides (119), Lotus leaves polysaccharides (57), and Aloe vera polysaccharides (141).

##### 3.2.4.3 NF-κB signaling pathway

The NF-κB transcription factor pathway holds a pivotal role in the regulation of inflammatory diseases and immune responses (142). NF-κB is particularly instrumental in orchestrating immunological responses and governing the polarization of M1 macrophages. The target genes under the influence of NF-κB encompass IL-1, IL-2, IL-6, IL-8, IL-12, and TNF-α. It has been demonstrated that inhibiting IKKβ in tumor-associated macrophages leads to increased expression of the antitumor cytokine IL-12 and inducible nitric oxide synthase, facilitating the transition of macrophage phenotype from M2 to M1 (143). Plant extracts and isolated compounds from numerous families directly target the NF-κB signaling cascade at a molecular level. Examples of plant polysaccharides that activate the NF-κB signaling pathway and foster M1 macrophage polarization are listed below: Crocus sativus polysaccharide (144), and Pleurotus ostreatus polysaccharides (145).

##### 3.2.4.4 JAK/STAT signaling pathways

The Janus kinase (JAK)-signal converter and activator of transcription (STAT) pathway (JAK/STAT) is activated by cytokines. Following STAT1-initiated transcription of M1 macrophage-typical genes, pro-inflammatory cytokines are released (146). The transcription factor STAT3, on the other hand, is involved in both development and tissue homeostasis. It has been found in multiple investigations that STAT3 activation can convert macrophages into M2-type (147, 148). A comprehensive analysis of the molecular mechanisms of macrophage polarization was carried out by Guo et al., who discovered that BRP regulates TAMs polarization via the STAT signaling pathway. Specifically, BRP controls M1 and M2 polarization by increasing STAT1 activation and decreasing STAT3 and STAT6 activation (149). Li et al. found that IAPS-2 polysaccharide has antitumor effects by inhibiting the phosphorylation of STAT3 in RAW 264.7 cells and S180 tumor tissues, while significantly increasing the phosphorylation of STAT1 (78).

Together, these mechanisms contribute to the regulation of macrophage polarization by natural plant-derived polysaccharides. It should be mentioned that the exact processes may differ based on the polysaccharide and the cellular environment. The signaling pathways and their molecular interactions by which natural plant-derived polysaccharides regulate macrophage polarization need to be further investigated.

## 4 Clinical translation and application

The development of natural products has been an important direction in antitumor drug discovery and research. This paper reviews some plant-derived crude and pure polysaccharides with clinical applications or ongoing clinical trials, aiming to provide new insights into anticancer immunotherapy. The clinical applications of four natural plant polysaccharides are summarized primarily in **Table 2**.

**Table 2:** A review of clinical studies on plant polysaccharides.

Study model	Therapeutics	Treatment target	Mechanism	Ref.
Astragalus membranaceus	Combined with immune Checkpoint Inhibitors	NSCLC	Reduce PD-L1 expression in TME; activate and proliferate tumor-specific T cells in TME	(82)
	CCRT	HNSCC	Activate CCRT-associated AEs and deterioration in QoL	(83)
	Combined with cisplatin	nasopharyngeal carcinoma	Enhance the anti-proliferative and apoptotic effect of cisplatin by modulating expression of Bax/Bcl-2 ratio and caspases	(84)
	Combined with Apatinib	gastric cancer	Inhibit AKT signalling pathway	(85)
RG-I Pectic Polysaccharides			Enhance phagocytic activity and stimulates cytokine secretion	(86)
EPS-EPO VIIa	Combined with chemotherapy	gastric cancer	Reduce chemotherapy-induced leukopenia	(87)
Belapectin	combined with anti-PD-1 (pembrolizumab)	MM and HNSCC	Enhance anti-tumor immunity by enhancing CD8+ T-cells and repolarize M2→M1 macrophages	(88)

### 4.1 *Astragalus* polysaccharide

Preclinical studies and clinical trials have demonstrated the antitumor effects of APS (92, 150). The anti-tumor effects of APS mainly include three aspects: first, they can improve the efficacy of chemotherapeutic drugs; second, they inhibit tumor cell proliferation and promote apoptosis; and third, they play an anti-tumor role through immune mechanisms (151).

APS can induce to overcome the inhibition of cyclophosphamide, promote the proliferation of lymphocytes, increase the serum antibody gradient, and enhance the ability of vaccine antigens thus widely used in clinics (42). Kong et al. reviewed the clinical trials and laboratory studies of APS and evaluated the potential feasibility of APS for use in combination with immunotherapy in the treatment of tumors (150). They noticed that APS can regulate immune cells, such as macrophages and NK cells, through cytokines and signaling pathways. Additionally, it is involved in the immune checkpoint inhibitor signaling pathway. Immune checkpoint inhibitors (ICIs) that can activate and multiply tumor-specific T cells in TME include PD-1 and CTLA-4 inhibitors. Neutrophil-to-lymphocyte ratio (NLR) is used as a prognostic indicator in immunotherapy-treated cancer patients. Recent research indicates that patients with NSCLC who have elevated NLR are more likely to have side effects and have lower survival rates (152, 153). PG2, a polysaccharide extracted from *Astragalus membranaceus*, as a prescription drug reduces the index NLR in patients with advanced lung cancer treated with a combination of ICIs (82). This finding suggests that APS could be used in combination with immunotherapy to treat tumors (150).

Guo et al. conducted a clinical trial with 136 patients to examine the efficacy and safety of administering APS along with vinorelbine and cisplatin (VC) for advanced NSCLC. The results demonstrated that compared to patients treated with VC alone, APS combined with VC treatment led to a better quality of survival (154). In a study performed by Hsieh et al., the effect of PG2 injection on concurrent chemoradiation therapy (CCRT)-related adverse Events (AEs) and patient adherence to treatment were investigated. The results showed that PG2 has a safety profile and has the potential to ameliorate the impact of AEs in advanced head and neck squamous cell carcinoma (HNSCC) under CCRT (83). In addition to enhancing chemotherapy’s effectiveness against NSCLC and HNSCC, APs have shown equal effectiveness in preclinical investigations against nasopharyngeal cancers (84), gastric (85), and ovarian malignancies respectively [132,140].

### 4.2 Belapectin

Proteins known as lectins bind carbohydrates and are members of the non-integrin β-galactoside-binding lectin family 6. Galactose lectin is an intracellular protein localized mainly in the cytoplasm and nucleus (155). Previous research has demonstrated that galectins have a significant role in the pathophysiology of cancer, fibrosis, and inflammation (156, 157). Galactose lectin-3 (Gal-3) is the most prominent galactose lectin secreted in disease states. Gal-3: this protein increases M2 polarization and macrophage infiltration, inhibits TCR signaling, and triggers T cell death to cause tumor-induced immunosuppression (158). Gal-3 is also upregulated by a number of cancers, and this is linked to a bad prognosis (159, 160). Several natural polysaccharides, Belapectin (GR-MD-02), Modified Citrus Pectin (MCP, PectaSol-C), and Davanat (GM-CT-01), are carbohydrate inhibitors of galactoglucan lectins (88, 161, 162). Of these, GR-MD-02 is currently being actively conducted and evaluated in various stages of clinical trials (163–165).

TCR-mediated signaling is essential for increasing effector T-cell responses to treatment with agonist anti-ox40 monoclonal antibody (aOX40) to maintain antitumor immunity (166). Sturgill et al. validated that belapectin synergizes with an agonist anti-OX40 antibody (aOX40) to promote tumor regression and improve survival by using hormonal (MCA-205 sarcoma, 4T1 breast cancer, TRAMP-C1 prostate adenocarcinoma) mice (167). Additionally, PD-1/PD-L1 involvement and overexpression of Gal-3 are key mechanisms of tumor-induced immunosuppression that contribute to immunotherapy resistance (168, 169). The researchers assessed the role of immunization in patients with metastatic melanoma (MM) and head and neck squamous cell carcinoma (HNSCC) by combining GR-MD-02) with anti-PD-1 (pembrolizumab) (88). The results of the phase I clinical trial found that the combination therapy of beraplanin + pembrolizumab was active against MM and HNSCC, and that dual blockade of PD-L1 and Gal-3 enhanced anti-tumor immunity by enhancing CD8+ T-cells, reducing MDSCs, and repolarizing M2→M1 macrophages (88).

### 4.3 Other polysaccharides

In a prospective study conducted by Melchart et al., EPS-EPO VIIa, a polysaccharide component isolated from *Echinacea purpurea herb* was shown to attenuate the adverse effects of chemotherapy in patients with advanced gastric cancer, but the exact mechanism remains to be investigated (87). Pectin polysaccharides rich in RG-I structure from bell peppers and carrots were proposed by Mckay et al. (86). Its ability to enhance innate immune responsiveness has been demonstrated in a series of preclinical and clinical studies to help boost immunity against infections.

In conclusion, combining chemotherapy with biological response modifiers offers a novel strategy for counteracting chemotherapy’s immunosuppressive effects; however, there are still obstacles to overcome in the clinical translation of plant polysaccharides, which are naturally occurring biological response modifiers. One of the biggest problems with clinical research is the scarcity of pure chemicals and well described extracts; therefore, many more defined extracts of active compounds will be needed for future clinical trials. Second, there has to be research into both clinical and experimental settings to establish whether polysaccharides increase cancer risk. Given the toxicity of many plant derivatives, it is important to choose the safest dosage of medication and take precautions to reduce the likelihood of adverse effects.

## 5 Discussion

In addition to conventional approaches such as surgery, chemotherapy, targeted therapy, and radiotherapy, immunotherapy has emerged as a cornerstone in standard cancer care. Macrophages, key components of immune effector cells, exert either pro- or anti-tumor effects by modulating their polarization in response to the tumor microenvironment. This notable plasticity presents opportunities for the depletion and repolarization of TAMs. Plant-derived polysaccharide molecules, originating from sources such as plants, algae, and fungi, are identified as potent immunomodulators in this review. These compounds activate innate immune responses in macrophages, effectively suppressing malignancies. Furthermore, plant polysaccharides have demonstrated the ability to enhance radiation sensitization, augment the efficacy of vaccinations, and serve as effective adjuvants. A large number of studies have demonstrated the ability of natural plant polysaccharides in cancer prevention and treatment. However, elucidating the direct targets and specific molecular mechanisms of natural plant polysaccharides still presents difficulties and challenges. First, the relationship between the structure and pharmacological activity of polysaccharides is unclear, and thus the study of immunomodulatory and anticancer mechanisms also poses challenges. In view of this, future research efforts may focus on identifying the optimal polysaccharide isolation technique, investigating the relationship between its chemical structure and biological activity, and exploring its role in cancer therapy. Secondly, the low bioavailability of natural polysaccharides is also a problem. Studies have shown that polysaccharides after oral administration are difficult to cross the biological barrier to act directly. Nanoparticles, characterized by favorable water solubility, stability, and biocompatibility, present a viable solution. Utilizing nanomaterials can enhance the bioavailability of polysaccharides, extending the effective duration of drugs within the body and mitigating potential side effects. In general, polysaccharides are not suitable as first-line medications in anti-cancer therapy, but only applied as adjuvant therapy. This is due to the unclear understanding of the mechanisms and targets underlying their natural pharmacological anti-tumor effects, thereby constraining their broader clinical applications.

In summary, this review provides a thorough analysis of the regulatory effects and mechanisms of plant polysaccharides on TAMs. Additionally, an analysis of potential opportunities for clinical translation of plant polysaccharides as immune adjuvants is presented. Further research on polysaccharides will lead to more efficient production and use of polysaccharide adjuvants.

## References

[1] Murciano-Goroff YR Warner AB Wolchok JD. The future of cancer immunotherapy: microenvironment-targeting combinations. Cell Res. (2020) 30:507–19. doi: 10.1038/s41422-020-0337-2 PMC7264181 32467593

[2] Jin M-Z Jin W-L. The updated landscape of tumor microenvironment and drug repurposing. Signal Transduct Target Ther. (2020) 5:166. doi: 10.1038/s41392-020-00280-x 32843638 PMC7447642

[3] Anderson NR Minutolo NG Gill S Klichinsky M. Macrophage-based approaches for cancer immunotherapy. Cancer Res. (2021) 81:1201–8. doi: 10.1158/0008-5472.CAN-20-2990 33203697

[4] DeNardo DG Ruffell B. Macrophages as regulators of tumour immunity and immunotherapy. Nat Rev Immunol. (2019) 19:369–82. doi: 10.1038/s41577-019-0127-6 PMC7339861 30718830

[5] Zhang Q Sioud M. Tumor-associated macrophage subsets: shaping polarization and targeting. Int J Mol Sci. (2023) 24:7493. doi: 10.3390/ijms24087493 37108657 PMC10138703

[6] Mantovani A Sozzani S Locati M Allavena P Sica A. Macrophage polarization: tumor-associated macrophages as a paradigm for polarized M2 mononuclear phagocytes. Trends Immunol. (2002) 23:549–55. doi: 10.1016/S1471-4906(02)02302-5 12401408

[7] Vasiliadou I Holen I. The role of macrophages in bone metastasis. J Bone Oncol. (2013) 2:158–66. doi: 10.1016/j.jbo.2013.07.002 PMC4723381 26909287

[8] Italiani P Boraschi D. From monocytes to M1/M2 macrophages: phenotypical vs. Functional differentiation. Front Immunol. (2014) 5:514. doi: 10.3389/fimmu.2014.00514 25368618 PMC4201108

[9] Loyher P-L Hamon P Laviron M Meghraoui-Kheddar A Goncalves E Deng Z. Macrophages of distinct origins contribute to tumor development in the lung. J Exp Med. (2018) 215:2536–53. doi: 10.1084/jem.20180534 PMC6170177 30201786

[10] Zhu Y Herndon JM Sojka DK Kim K-W Knolhoff BL Zuo C. Tissue-resident macrophages in pancreatic ductal adenocarcinoma originate from embryonic hematopoiesis and promote tumor progression. Immunity. (2017) 47:323–338.e6. doi: 10.1016/j.immuni.2017.07.014 28813661 PMC5578409

[11] Chen Z Feng X Herting CJ Garcia VA Nie K Pong WW. Cellular and molecular identity of tumor-associated macrophages in glioblastoma. Cancer Res. (2017) 77:2266–78. doi: 10.1158/0008-5472.CAN-16-2310 PMC5741820 28235764

[12] Pittet MJ Michielin O Migliorini D. Clinical relevance of tumour-associated macrophages. Nat Rev Clin Oncol. (2022) 19:402–21. doi: 10.1038/s41571-022-00620-6 35354979

[13] Qian B-Z Pollard JW. Macrophage diversity enhances tumor progression and metastasis. Cell. (2010) 141:39–51. doi: 10.1016/j.cell.2010.03.014 20371344 PMC4994190

[14] Ruffell B Coussens LM. Macrophages and therapeutic resistance in cancer. Cancer Cell. (2015) 27:462–72. doi: 10.1016/j.ccell.2015.02.015 PMC4400235 25858805

[15] Chen Y Jin H Song Y Huang T Cao J Tang Q. Targeting tumor-associated macrophages: A potential treatment for solid tumors. J Cell Physiol. (2021) 236:3445–65. doi: 10.1002/jcp.30139 33200401

[16] Tiwari A Trivedi R Lin S-Y. Tumor microenvironment: barrier or opportunity towards effective cancer therapy. J BioMed Sci. (2022) 29:83. doi: 10.1186/s12929-022-00866-3 36253762 PMC9575280

[17] Chen D Xie J Fiskesund R Dong W Liang X Lv J. Chloroquine modulates antitumor immune response by resetting tumor-associated macrophages toward M1 phenotype. Nat Commun. (2018) 9:873. doi: 10.1038/s41467-018-03225-9 29491374 PMC5830447

[18] Wang X Gao A Jiao Y Zhao Y Yang X. Antitumor effect and molecular mechanism of antioxidant polysaccharides from Salvia miltiorrhiza Bunge in human colorectal carcinoma LoVo cells. Int J Biol Macromol. (2018) 108:625–34. doi: 10.1016/j.ijbiomac.2017.12.006 29233711

[19] Wang A Liu Y Zeng S Liu Y Li W Wu D. Dietary plant polysaccharides for cancer prevention: role of immune cells and gut microbiota, challenges and perspectives. Nutrients. (2023) 15:3019. doi: 10.3390/nu15133019 37447345 PMC10347129

[20] Merecz-Sadowska A Sitarek P Śliwiński T Zajdel R. Anti-inflammatory activity of extracts and pure compounds derived from plants via modulation of signaling pathways, especially PI3K/AKT in macrophages. Int J Mol Sci. (2020) 21:9605. doi: 10.3390/ijms21249605 33339446 PMC7766727

[21] Chen L Huang G. Antitumor activity of polysaccharides: an overview. Curr Drug Targets. (2018) 19:89-96. doi: 10.2174/1389450118666170704143018 28676001

[22] Abdelaziz MH Abdelwahab SF Wan J Cai W Huixuan W Jianjun C. Alternatively activated macrophages; a double-edged sword in allergic asthma. J Transl Med. (2020) 18:58. doi: 10.1186/s12967-020-02251-w 32024540 PMC7003359

[23] Franklin RA Liao W Sarkar A Kim MV Bivona MR Liu K. The cellular and molecular origin of tumor-associated macrophages. Science. (2014) 344:921–5. doi: 10.1126/science.1252510 PMC4204732 24812208

[24] Mantovani A Marchesi F Malesci A Laghi L Allavena P. Tumor-associated macrophages as treatment targets in oncology. Nat Rev Clin Oncol. (2017) 14:399–416. doi: 10.1038/nrclinonc.2016.217 28117416 PMC5480600

[25] Wang S Liu G Li Y Pan Y. Metabolic reprogramming induces macrophage polarization in the tumor microenvironment. Front Immunol. (2022) 13:840029. doi: 10.3389/fimmu.2022.840029 35874739 PMC9302576

[26] Ghafouri-Fard S Abak A Tavakkoli Avval S Shoorei H Taheri M Samadian M. The impact of non-coding RNAs on macrophage polarization. Biomed Pharmacother. (2021) 142:112112. doi: 10.1016/j.biopha.2021.112112 34449319

[27] Schlundt C Fischer H Bucher CH Rendenbach C Duda GN Schmidt-Bleek K. The multifaceted roles of macrophages in bone regeneration: A story of polarization, activation and time. Acta Biomater. (2021) 133:46–57. doi: 10.1016/j.actbio.2021.04.052 33974949

[28] Colin S Chinetti-Gbaguidi G Staels B. Macrophage phenotypes in atherosclerosis. Immunol Rev. (2014) 262:153–66. doi: 10.1111/imr.12218 25319333

[29] Boutilier AJ Elsawa SF. Macrophage polarization states in the tumor microenvironment. Int J Mol Sci. (2021) 22:6995. doi: 10.3390/ijms22136995 34209703 PMC8268869

[30] Ghafouri-Fard S Abak A Tavakkoli Avval S Shoorei H Taheri M Samadian M. The impact of non-coding RNAs on macrophage polarization. BioMed Pharmacother. (2021) 142:112112. doi: 10.1016/j.biopha.2021.112112 34449319

[31] Noy R Pollard JW. Tumor-associated macrophages: from mechanisms to therapy. Immunity. (2014) 41:49–61. doi: 10.1016/j.immuni.2014.06.010 25035953 PMC4137410

[32] Liu Y-C Yu M-M Chai Y-F Shou S-T. Sialic acids in the immune response during sepsis. Front Immunol. (2017) 8:1601. doi: 10.3389/fimmu.2017.01601 29209331 PMC5702289

[33] Logtenberg MEW Scheeren FA Schumacher TN. The CD47-SIRPα Immune checkpoint. Immunity. (2020) 52:742–52. doi: 10.1016/j.immuni.2020.04.011 PMC7340539 32433947

[34] Umiker B Hashambhoy-Ramsay Y Smith J Rahman T Mueller A Davidson R. Inhibition of LILRB2 by a novel blocking antibody designed to reprogram immunosuppressive macrophages to drive T-cell activation in tumors. Mol Cancer Ther. (2023) 22:471–84. doi: 10.1158/1535-7163.MCT-22-0351 36780212

[35] Dong Q Zhang S Zhang H Sun J Lu J Wang G. MARCO is a potential prognostic and immunotherapy biomarker. Int Immunopharmacol. (2023) 116:109783. doi: 10.1016/j.intimp.2023.109783 36773567

[36] Mantovani A Bonecchi R. One clever macrophage checkpoint. Clin Cancer Res Off J Am Assoc Cancer Res. (2019) 25:3202–4. doi: 10.1158/1078-0432.CCR-19-0483 PMC7174017 30936124

[37] Sugimura-Nagata A Koshino A Inoue S Matsuo-Nagano A Komura M Riku M. Expression and prognostic significance of CD47-SIRPA macrophage checkpoint molecules in colorectal cancer. Int J Mol Sci. (2021) 22:2690. doi: 10.3390/ijms22052690 33799989 PMC7975978

[38] Barkal AA Brewer RE Markovic M Kowarsky M Barkal SA Zaro BW. CD24 signalling through macrophage Siglec-10 is a target for cancer immunotherapy. Nature. (2019) 572:392–6. doi: 10.1038/s41586-019-1456-0 PMC6697206 31367043

[39] Shen W Shi P Dong Q Zhou X Chen C Sui X. Discovery of a novel dual-targeting D-peptide to block CD24/Siglec-10 and PD-1/PD-L1 interaction and synergize with radiotherapy for cancer immunotherapy. J Immunother Cancer. (2023) 11:e007068. doi: 10.1136/jitc-2023-007068 37344099 PMC10314633

[40] Zha Z Wang S-Y Chu W Lv Y Kan H Chen Q. Isolation, purification, structural characterization and immunostimulatory activity of water-soluble polysaccharides from Lepidium meyenii. Phytochemistry. (2018) 147:184–93. doi: 10.1016/j.phytochem.2018.01.006 29353155

[41] Ying Y Hao W. Immunomodulatory function and anti-tumor mechanism of natural polysaccharides: A review. Front Immunol. (2023) 14:1147641. doi: 10.3389/fimmu.2023.1147641 36969152 PMC10035574

[42] Guo L Liu J Hu Y Wang D Li Z Zhang J. Astragalus polysaccharide and sulfated epimedium polysaccharide synergistically resist the immunosuppression. Carbohydr Polym. (2012) 90:1055–60. doi: 10.1016/j.carbpol.2012.06.042 22840039

[43] Gao Z Liu K Tian W Wang H Liu Z Li Y. Effects of selenizing angelica polysaccharide and selenizing garlic polysaccharide on immune function of murine peritoneal macrophage. Int Immunopharmacol. (2015) 27:104–9. doi: 10.1016/j.intimp.2015.04.052 25962819

[44] Cai G Wu C Zhu T Peng S Xu S Hu Y. Structure of a Pueraria root polysaccharide and its immunoregulatory activity on T and B lymphocytes, macrophages, and immunosuppressive mice. Int J Biol Macromol. (2023) 230:123386. doi: 10.1016/j.ijbiomac.2023.123386 36702224

[45] Dong Q Yao J Fang J Ding K. Structural characterization and immunological activity of two cold-water extractable polysaccharides from Cistanche deserticola Y. C. Ma. Carbohydr Res. (2007) 342:1343–9. doi: 10.1016/j.carres.2007.03.017 17442280

[46] Chen Y Li H Li M Niu S Wang J Shao H. Salvia miltiorrhiza polysaccharide activates T Lymphocytes of cancer patients through activation of TLRs mediated -MAPK and -NF-κB signaling pathways. J Ethnopharmacol. (2017) 200:165–73. doi: 10.1016/j.jep.2017.02.029 28232127

[47] Wu M Li W Zhang Y Shi L Xu Z Xia W. Structure characteristics, hypoglycemic and immunomodulatory activities of pectic polysaccharides from Rosa setate x Rosa rugosa waste. Carbohydr Polym. (2021) 253:117190. doi: 10.1016/j.carbpol.2020.117190 33278967

[48] Li W Hu X Wang S Jiao Z Sun T Liu T. Characterization and anti-tumor bioactivity of astragalus polysaccharides by immunomodulation. Int J Biol Macromol. (2020) 145:985–97. doi: 10.1016/j.ijbiomac.2019.09.189 31669273

[49] Wang N Zhang X Wang S Guo Q Li Z Liu H. Structural characterisation and immunomodulatory activity of polysaccharides from white asparagus skin. Carbohydr Polym. (2020) 227:115314. doi: 10.1016/j.carbpol.2019.115314 31590844

[50] Wang M Liu Y Qiang M Wang J. Structural elucidation of a pectin-type polysaccharide from Hovenia dulcis peduncles and its proliferative activity on RAW264.7 cells. Int J Biol Macromol. (2017) 104:1246–53. doi: 10.1016/j.ijbiomac.2017.07.004 28715863

[51] Yan J-K Wang C Yu Y-B Wu L-X Chen T-T Wang Z-W. Physicochemical characteristics and in *vitro* biological activities of polysaccharides derived from raw garlic (Allium sativum L.) bulbs via three-phase partitioning combined with gradient ethanol precipitation method. Food Chem. (2021) 339:128081. doi: 10.1016/j.foodchem.2020.128081 33152874

[52] Cao W Li X-Q Wang X Li T Chen X Liu S-B. Characterizations and anti-tumor activities of three acidic polysaccharides from Angelica sinensis (Oliv.) Diels. Int J Biol Macromol. (2010) 46:115–22. doi: 10.1016/j.ijbiomac.2009.11.005 19941888

[53] Zhang M Wang G Lai F Wu H. Structural characterization and immunomodulatory activity of a novel polysaccharide from *lepidium meyenii* . J Agric Food Chem. (2016) 64:1921–31. doi: 10.1021/acs.jafc.5b05610 26883006

[54] Zhang M Wu W Ren Y Li X Tang Y Min T. Structural characterization of a novel polysaccharide from *lepidium meyenii* (Maca) and analysis of its regulatory function in macrophage polarization in vitro. J Agric Food Chem. (2017) 65:1146–57. doi: 10.1021/acs.jafc.6b05218 28117590

[55] Leung MYK. Chemical and biological characterization of a polysaccharide biological response modifier from Aloe vera L. var. chinensis (Haw.) Berg. Glycobiology. (2004) 14:501–10. doi: 10.1093/glycob/cwh050 14739149

[56] Hou T Guo S Liu Z Lin H Song Y Li Q. Novel pectic polysaccharides isolated from immature honey pomelo fruit with high immunomodulatory activity. Molecules. (2022) 27:8573. doi: 10.3390/molecules27238573 36500662 PMC9739730

[57] Song Y-R Han A-R Lim T-G Lee E-J Hong H-D. Isolation, purification, and characterization of novel polysaccharides from lotus (Nelumbo nucifera) leaves and their immunostimulatory effects. Int J Biol Macromol. (2019) 128:546–55. doi: 10.1016/j.ijbiomac.2019.01.131 30685309

[58] Nie C Zhu P Ma S Wang M Hu Y. Purification, characterization and immunomodulatory activity of polysaccharides from stem lettuce. Carbohydr Polym. (2018) 188:236–42. doi: 10.1016/j.carbpol.2018.02.009 29525161

[59] Zhan Q Wang Q Lin R He P Lai F Zhang M. Structural characterization and immunomodulatory activity of a novel acid polysaccharide isolated from the pulp of Rosa laevigata Michx fruit. Int J Biol Macromol. (2020) 145:1080–90. doi: 10.1016/j.ijbiomac.2019.09.201 31730989

[60] Jeon H Oh S Kum E Seo S Park Y Kim G. Immunomodulatory effects of an aqueous extract of black radish on mouse macrophages via the TLR2/4-mediated signaling pathway. Pharmaceuticals. (2022) 15:1376. doi: 10.3390/ph15111376 36355548 PMC9697478

[61] Lin P Chen L Huang X Xiao F Fu L Jing D. Structural characteristics of polysaccharide GP2a in gardenia jasminoides and its immunomodulatory effect on macrophages. Int J Mol Sci. (2022) 23:11279. doi: 10.3390/ijms231911279 36232580 PMC9569544

[62] Qu D Lian S Hu H Sun W Si H. Characterization and macrophages immunomodulatory activity of two water-soluble polysaccharides from Abrus cantoniensis. Front Nutr. (2022) 9:969512. doi: 10.3389/fnut.2022.969512 36071932 PMC9441930

[63] Yang Y Yin X Zhang D Lu J Wang X. Isolation, structural characterization and macrophage activation activity of an acidic polysaccharide from raspberry pulp. Molecules. (2022) 27:1674. doi: 10.3390/molecules27051674 35268775 PMC8911918

[64] Chen Z Soo MY Srinivasan N Tan BKH Chan SH. Activation of macrophages by polysaccharide-protein complex from *Lycium barbarum* L. Phytother Res. (2009) 23:1116–22. doi: 10.1002/ptr.2757 19170138

[65] Yang Y Yin X Zhang D Zhang B Lu J Wang X. Structural characteristics, antioxidant, and immunostimulatory activities of an acidic polysaccharide from raspberry pulp. Molecules. (2022) 27:4385. doi: 10.3390/molecules27144385 35889258 PMC9318036

[66] Yang X Wu Y Zhang C Fu S Zhang J Fu C. Extraction, structural characterization, and immunoregulatory effect of a polysaccharide fraction from Radix Aconiti Lateralis Preparata (Fuzi). Int J Biol Macromol. (2020) 143:314–24. doi: 10.1016/j.ijbiomac.2019.11.208 31786293

[67] Fan S Wang Y Zhang Y Wu Y Chen X. Achyranthes bidentata polysaccharide activates nuclear factor-kappa B and promotes cytokine production in J774A.1 cells through TLR4/myD88 signaling pathway. Front Pharmacol. (2021) 12:753599. doi: 10.3389/fphar.2021.753599 34658894 PMC8517515

[68] Yu Y Shen M Wang Z Wang Y Xie M Xie J. Sulfated polysaccharide from Cyclocarya paliurus enhances the immunomodulatory activity of macrophages. Carbohydr Polym. (2017) 174:669–76. doi: 10.1016/j.carbpol.2017.07.009 28821118

[69] Pan G Xie Z Huang S Tai Y Cai Q Jiang W. Immune-enhancing effects of polysaccharides extracted from Lilium lancifolium Thunb. Int Immunopharmacol. (2017) 52:119–26. doi: 10.1016/j.intimp.2017.08.030 28898768

[70] Ando I Tsukumo Y Wakabayashi T Akashi S Miyake K Kataoka T. Safflower polysaccharides activate the transcription factor NF-nB via Toll-like receptor 4 and induce cytokine production by macrophages. Int Immunopharmacol. (2002) 2: 1155-62. doi: 10.1016/S1567-5769(02)00076-0 12349952

[71] Zhao T Feng Y Li J Mao R Zou Y Feng W. Schisandra polysaccharide evokes immunomodulatory activity through TLR 4-mediated activation of macrophages. Int J Biol Macromol. (2014) 65:33–40. doi: 10.1016/j.ijbiomac.2014.01.018 24418335

[72] Cheng A Wan F Wang J Jin Z Xu X. Macrophage immunomodulatory activity of polysaccharides isolated from Glycyrrhiza uralensis fish. Int Immunopharmacol. (2008) 8:43–50. doi: 10.1016/j.intimp.2007.10.006 18068099

[73] Han SB Park SH Lee KH Lee CW Lee SH Kim HC. Polysaccharide isolated from the radix of Platycodon grandiflorum selectively activates B cells and macrophages but not T cells. Int Immunopharmacol. (2001) 1:1969–78. doi: 10.1016/S1567-5769(01)00124-2 11606028

[74] Wei W Xiao H-T Bao W-R Ma D-L Leung C-H Han X-Q. TLR-4 may mediate signaling pathways of Astragalus polysaccharide RAP induced cytokine expression of RAW264.7 cells. J Ethnopharmacol. (2016) 179:243–52. doi: 10.1016/j.jep.2015.12.060 26743224

[75] Long T Liu Z Shang J Zhou X Yu S Tian H. Polygonatum sibiricum polysaccharides play anti-cancer effect through TLR4-MAPK/NF-κB signaling pathways. Int J Biol Macromol. (2018) 111:813–21. doi: 10.1016/j.ijbiomac.2018.01.070 29343453

[76] Sun Y Diao F Niu Y Li X Zhou H Mei Q. Apple polysaccharide prevents from colitis-associated carcinogenesis through regulating macrophage polarization. Int J Biol Macromol. (2020) 161:704–11. doi: 10.1016/j.ijbiomac.2020.06.121 32544579

[77] Chen M Li Y Liu Z Qu Y Zhang H Li D. Exopolysaccharides from a *Codonopsis pilosula* endophyte activate macrophages and inhibit cancer cell proliferation and migration. Thorac Cancer. (2018) 9:630–9. doi: 10.1111/1759-7714.12630 PMC5928371 29577649

[78] Li Q Hao Z Hong Y He W Zhao W. Reprogramming tumor associated macrophage phenotype by a polysaccharide from ilex asprella for sarcoma immunotherapy. Int J Mol Sci. (2018) 19:3816. doi: 10.3390/ijms19123816 30513582 PMC6320939

[79] Xiong L Ouyang K-H Jiang Y Yang Z-W Hu W-B Chen H. Chemical composition of Cyclocarya paliurus polysaccharide and inflammatory effects in lipopolysaccharide-stimulated RAW264.7 macrophage. Int J Biol Macromol. (2018) 107:1898–907. doi: 10.1016/j.ijbiomac.2017.10.055 29032210

[80] Wang M Yang X-B Zhao J-W Lu C-J Zhu W. Structural characterization and macrophage immunomodulatory activity of a novel polysaccharide from Smilax glabra Roxb. Carbohydr Polym. (2017) 156:390–402. doi: 10.1016/j.carbpol.2016.09.033 27842838

[81] Xie X-D Tang M Yi S-L He Y Chen S-Y Zhao Y. Polysaccharide of Asparagus cochinchinensis (Lour.) Merr regulates macrophage immune response and epigenetic memory through TLR4-JNK/p38/ERK signaling pathway and histone modification. Phytomedicine Int J Phytother Phytopharm. (2024) 124:155294. doi: 10.1016/j.phymed.2023.155294 38176271

[82] Tsao SM Wu TC Chen J Chang F Tsao T. Astragalus polysaccharide injection (PG2) normalizes the neutrophil-to-lymphocyte ratio in patients with advanced lung cancer receiving immunotherapy. Integr Cancer Ther. (2021) 20:153473542199525. doi: 10.1177/1534735421995256 PMC7890706 33583212

[83] Hsieh C-H Lin C-Y Hsu C-L Fan K-H Huang S-F Liao C-T. Incorporation of Astragalus polysaccharides injection during concurrent chemoradiotherapy in advanced pharyngeal or laryngeal squamous cell carcinoma: preliminary experience of a phase II double-blind, randomized trial. J Cancer Res Clin Oncol. (2020) 146:33–41. doi: 10.1007/s00432-019-03033-8 31728618 PMC11804622

[84] Zhou Z Meng M Ni H. Chemosensitizing effect of astragalus polysaccharides on nasopharyngeal carcinoma cells by inducing apoptosis and modulating expression of bax/bcl-2 ratio and caspases. Med Sci Monit. (2017) 23:462–9. doi: 10.12659/MSM.903170 PMC5291085 28124680

[85] Wu J Yu J Wang J Zhang C Shang K Yao X. Astragalus polysaccharide enhanced antitumor effects of Apatinib in gastric cancer AGS cells by inhibiting AKT signalling pathway. BioMed Pharmacother. (2018) 100:176–83. doi: 10.1016/j.biopha.2018.01.140 29428665

[86] McKay S Oranje P Helin J Koek JH Kreijveld E van den Abbeele P. Development of an affordable, sustainable and efficacious plant-based immunomodulatory food ingredient based on bell pepper or carrot RG-I pectic polysaccharides. Nutrients. (2021) 13:963. doi: 10.3390/nu13030963 33809720 PMC8002328

[87] Melchart D Clemm C Weber B Draczynski T Worku F Linde K. Polysaccharides isolated from EChinacea purpurea herba cell cultures to counteract undesired effects of chemotherapy–a pilot study. Phytother Res PTR. (2002) 16:138–42. doi: 10.1002/ptr.888 11933115

[88] Curti BD Koguchi Y Leidner RS Rolig AS Sturgill ER Sun Z. Enhancing clinical and immunological effects of anti-PD-1 with belapectin, a galectin-3 inhibitor. J Immunother Cancer. (2021) 9:e002371. doi: 10.1136/jitc-2021-002371 33837055 PMC8043038

[89] Qi J Kim SM. Characterization and immunomodulatory activities of polysaccharides extracted from green alga Chlorella ellipsoidea. Int J Biol Macromol. (2017) 95:106–14. doi: 10.1016/j.ijbiomac.2016.11.039 27856321

[90] Wu X-M Tu P-F. Isolation and characterization of α-(1→6)-glucans from *Cistanche deserticola* . J Asian Nat Prod Res. (2005) 7:823–8. doi: 10.1080/10286020410001721087 16308198

[91] Nie C Zhu P Wang M Ma S Wei Z. Optimization of water-soluble polysaccharides from stem lettuce by response surface methodology and study on its characterization and bioactivities. Int J Biol Macromol. (2017) 105:912–23. doi: 10.1016/j.ijbiomac.2017.07.125 28743571

[92] Li C Liu Y Zhang Y Li J Lai J. Astragalus polysaccharide: a review of its immunomodulatory effect. Arch Pharm Res. (2022) 45:367–89. doi: 10.1007/s12272-022-01393-3 35713852

[93] Bamodu OA Kuo K-T Wang C-H Huang W-C Wu ATH Tsai J-T. Astragalus polysaccharides (PG2) enhances the M1 polarization of macrophages, functional maturation of dendritic cells, and T cell-mediated anticancer immune responses in patients with lung cancer. Nutrients. (2019) 11:2264. doi: 10.3390/nu11102264 31547048 PMC6836209

[94] Yin J-Y. Separation, structure characterization, conformation and immunomodulating effect of a hyperbranched heteroglycan from Radix Astragali. Carbohydr Polym. (2012) 87: 667-675. doi: 10.1016/j.carbpol.2011.08.045 34663019

[95] Bader JE Enos RT Velázquez KT Carson MS Nagarkatti M Nagarkatti PS. Macrophage depletion using clodronate liposomes decreases tumorigenesis and alters gut microbiota in the AOM/DSS mouse model of colon cancer. Am J Physiol Gastrointest Liver Physiol. (2018) 314:G22–31. doi: 10.1152/ajpgi.00229.2017 PMC5866374 29025731

[96] Xie H Fang J Farag MA Li Z Sun P Shao P. Dendrobium officinale leaf polysaccharides regulation of immune response and gut microbiota composition in cyclophosphamide-treated mice. Food Chem X. (2022) 13:100235. doi: 10.1016/j.fochx.2022.100235 35499019 PMC9039934

[97] Briukhovetska D Dörr J Endres S Libby P Dinarello CA Kobold S. Interleukins in cancer: from biology to therapy. Nat Rev Cancer. (2021) 21:481–99. doi: 10.1038/s41568-021-00363-z PMC8173513 34083781

[98] Im S-A Lee Y-R Lee Y-H Oh S-T Gerelchuluun T Kim B-H. Synergistic activation of monocytes by polysaccharides isolated from Salicornia herbacea and interferon-gamma. J Ethnopharmacol. (2007) 111:365–70. doi: 10.1016/j.jep.2006.11.027 17204386

[99] Wang H Yung MMH Ngan HYS Chan KKL Chan DW. The impact of the tumor microenvironment on macrophage polarization in cancer metastatic progression. Int J Mol Sci. (2021) 22:6560. doi: 10.3390/ijms22126560 34207286 PMC8235734

[100] Xuan W Qu Q Zheng B Xiong S Fan G-H. The chemotaxis of M1 and M2 macrophages is regulated by different chemokines. J Leukoc Biol. (2015) 97:61–9. doi: 10.1189/jlb.1A0314-170R 25359998

[101] Ross JL Chen Z Herting CJ Grabovska Y Szulzewsky F Puigdelloses M. Platelet-derived growth factor beta is a potent inflammatory driver in paediatric high-grade glioma. Brain. (2021) 144:53–69. doi: 10.1093/brain/awaa382 33300045 PMC7954387

[102] Nie Y Huang H Guo M Chen J Wu W Li W. Breast phyllodes tumors recruit and repolarize tumor-associated macrophages via secreting CCL5 to promote Malignant progression, which can be inhibited by CCR5 inhibition therapy. Clin Cancer Res. (2019) 25:3873–86. doi: 10.1158/1078-0432.CCR-18-3421 30890553

[103] Yin X Han S Song C Zou H Wei Z Xu W. Metformin enhances gefitinib efficacy by interfering with interactions between tumor-associated macrophages and head and neck squamous cell carcinoma cells. Cell Oncol. (2019) 42:459–75. doi: 10.1007/s13402-019-00446-y PMC12994286 31001733

[104] Korbecki J Olbromski M Dzięgiel P. CCL18 in the progression of cancer. Int J Mol Sci. (2020) 21:7955. doi: 10.3390/ijms21217955 33114763 PMC7663205

[105] Liu C Yao Z Wang J Zhang W Yang Y Zhang Y. Macrophage-derived CCL5 facilitates immune escape of colorectal cancer cells via the p65/STAT3-CSN5-PD-L1 pathway. Cell Death Differ. (2020) 27:1765–81. doi: 10.1038/s41418-019-0460-0 PMC7244707 31802034

[106] Qin R Ren W Ya G Wang B He J Ren S. Role of chemokines in the crosstalk between tumor and tumor-associated macrophages. Clin Exp Med. (2022) 23:1359–73. doi: 10.1007/s10238-022-00888-z PMC10460746 36173487

[107] Burns JJ Zhao L Taylor EW Spelman K. The influence of traditional herbal formulas on cytokine activity. Toxicology. (2010) 278:140–59. doi: 10.1016/j.tox.2009.09.020 19818374

[108] Van den Bossche J Baardman J Otto NA van der Velden S Neele AE van den Berg SM. Mitochondrial dysfunction prevents repolarization of inflammatory macrophages. Cell Rep. (2016) 17:684–96. doi: 10.1016/j.celrep.2016.09.008 27732846

[109] Mills CD Lenz LL Harris RA. A breakthrough: macrophage-directed cancer immunotherapy. Cancer Res. (2016) 76:513–6. doi: 10.1158/0008-5472.CAN-15-1737 PMC4738030 26772756

[110] Zhou L Liu Z Wang Z Yu S Long T Zhou X. Astragalus polysaccharides exerts immunomodulatory effects via TLR4-mediated MyD88-dependent signaling pathway in *vitro* and in *vivo* . Sci Rep. (2017) 7:44822. doi: 10.1038/srep44822 28303957 PMC5355992

[111] Schepetkin IA Faulkner CL Nelson-Overton LK Wiley JA Quinn MT. Macrophage immunomodulatory activity of polysaccharides isolated from Juniperus scopolorum. Int Immunopharmacol. (2005) 5:1783–99. doi: 10.1016/j.intimp.2005.05.009 16275615

[112] Duan T Du Y Xing C Wang HY Wang R-F. Toll-like receptor signaling and its role in cell-mediated immunity. Front Immunol. (2022) 13:812774. doi: 10.3389/fimmu.2022.812774 35309296 PMC8927970

[113] Xiao Z Zhou W Zhang Y. "Fungal polysaccharides.," Advances in pharmacology. Elsevier (2020). p. 277–99. doi: 10.1016/bs.apha.2019.08.003 32089236

[114] Wei H Shi Y Yuan Z Huang Z Cai F Zhu J. Isolation, identification, and anti-inflammatory activity of polysaccharides of typha angustifolia. Biomacromolecules. (2021) 22:2451–9. doi: 10.1021/acs.biomac.1c00235 34024108

[115] Qiu Y Batool Z Liu R Sui G Sheng B Zheng X. Characterization and immunological activity of polysaccharides from Potentilla chinensis. Int J Biol Macromol. (2020) 165:683–90. doi: 10.1016/j.ijbiomac.2020.09.118 32961189

[116] Hsu H-Y Hua K-F Lin C-C Lin C-H Hsu J Wong C-H. Extract of reishi polysaccharides induces cytokine expression via TLR4-modulated protein kinase signaling pathways. J Immunol. (2004) 173:5989–99. doi: 10.4049/jimmunol.173.10.5989 15528333

[117] Tian H Liu Z Pu Y Bao Y. Immunomodulatory effects exerted by Poria Cocos polysaccharides via TLR4/TRAF6/NF-κB signaling in *vitro* and in *vivo* . BioMed Pharmacother. (2019) 112:108709. doi: 10.1016/j.biopha.2019.108709 30970514

[118] Schepetkin IA Quinn MT. Botanical polysaccharides: Macrophage immunomodulation and therapeutic potential. Int Immunopharmacol. (2006) 6:317–33. doi: 10.1016/j.intimp.2005.10.005 16428067

[119] Zhou S-F Cheng J Zhou Z-W Sheng H-P He L-J Fan X-W. An evidence-based update on the pharmacological activities and possible molecular targets of Lycium barbarum polysaccharides. Drug Des Devel Ther. (2014) 33:33-78. doi: 10.2147/DDDT.S72892 PMC4277126 25552899

[120] Cummings RD. The mannose receptor ligands and the macrophage glycome. Curr Opin Struct Biol. (2022) 75:102394. doi: 10.1016/j.sbi.2022.102394 35617912 PMC10243190

[121] Murgas P Cornejo FA Merino G Von Bernhardi R. SR-A regulates the inflammatory activation of astrocytes. Neurotox Res. (2014) 25:68–80. doi: 10.1007/s12640-013-9432-1 24114771

[122] Yang Y-S Jin X Li Q Chen Y-Y Chen F Zhang H. Superenhancer drives a tumor-specific splicing variant of MARCO to promote triple-negative breast cancer progression. Proc Natl Acad Sci U.S.A. (2022) 119:e2207201119. doi: 10.1073/pnas.2207201119 36343244 PMC9674263

[123] La Fleur L Botling J He F Pelicano C Zhou C He C. Targeting MARCO and IL37R on immunosuppressive macrophages in lung cancer blocks regulatory T cells and supports cytotoxic lymphocyte function. Cancer Res. (2021) 81:956–67. doi: 10.1158/0008-5472.CAN-20-1885 33293426

[124] Ding L Qian J Yu X Wu Q Mao J Liu X. Blocking MARCO+ tumor-associated macrophages improves anti-PD-L1 therapy of hepatocellular carcinoma by promoting the activation of STING-IFN type I pathway. Cancer Lett. (2024) 582:216568. doi: 10.1016/j.canlet.2023.216568 38065400

[125] Eisinger S Sarhan D Boura VF Ibarlucea-Benitez I Tyystjärvi S Oliynyk G. Targeting a scavenger receptor on tumor-associated macrophages activates tumor cell killing by natural killer cells. Proc Natl Acad Sci U.S.A. (2020) 117:32005–16. doi: 10.1073/pnas.2015343117 PMC7750482 33229588

[126] Arnaout MA. Structure and function of the leukocyte adhesion molecules CD11/CD18. Blood. (1990) 75:1037–50. doi: 10.1182/blood.V75.5.1037.1037 1968349

[127] Chan GC-F Chan WK Sze DM-Y. The effects of β-glucan on human immune and cancer cells. J Hematol OncolJ Hematol Oncol. (2009) 2:25. doi: 10.1186/1756-8722-2-25 19515245 PMC2704234

[128] Schütt C. CD14. Int J Biochem Cell Biol. (1999) 31:545–9. doi: 10.1016/S1357-2725(98)00153-8 10399315

[129] Goodridge HS Reyes CN Becker CA Katsumoto TR Ma J Wolf AJ. Activation of the innate immune receptor Dectin-1 upon formation of a ‘phagocytic synapse.’. Nature. (2011) 472:471–5. doi: 10.1038/nature10071 PMC3084546 21525931

[130] Ferwerda G Meyer-Wentrup F Kullberg B-J Netea MG Adema GJ. Dectin-1 synergizes with TLR2 and TLR4 for cytokine production in human primary monocytes and macrophages. Cell Microbiol. (2008) 10:2058–66. doi: 10.1111/j.1462-5822.2008.01188.x 18549457

[131] Gordon S. Pattern recognition receptors. Cell. (2002) 111:927–30. doi: 10.1016/S0092-8674(02)01201-1 12507420

[132] Qi Y Duan G Fan G Peng N. Effect of Lycium barbarum polysaccharides on cell signal transduction pathways. BioMed Pharmacother. (2022) 147:112620. doi: 10.1016/j.biopha.2022.112620 35032768

[133] Yin M Zhang Y Li H. Advances in research on immunoregulation of macrophages by plant polysaccharides. Front Immunol. (2019) 10:145. doi: 10.3389/fimmu.2019.00145 30804942 PMC6370632

[134] Mokhtari Y Pourbagheri-Sigaroodi A Zafari P Bagheri N Ghaffari SH Bashash D. Toll-like receptors (TLRs): An old family of immune receptors with a new face in cancer pathogenesis. J Cell Mol Med. (2021) 25:639–51. doi: 10.1111/jcmm.16214 PMC7812258 33336901

[135] Li B-X Li W-Y Tian Y-B Guo S-X Huang Y-M Xu D-N. Polysaccharide of *atractylodes macrocephala* koidz enhances cytokine secretion by stimulating the *TLR4–myD88–NF-* κ *B* signaling pathway in the mouse spleen. J Med Food. (2019) 22:937–43. doi: 10.1089/jmf.2018.4393 31448992

[136] Liu C Wang S Xiang Z Xu T He M Xue Q. The chemistry and efficacy benefits of polysaccharides from Atractylodes macrocephala Koidz. Front Pharmacol. (2022) 13:952061. doi: 10.3389/fphar.2022.952061 36091757 PMC9452894

[137] Kondoh K Nishida E. Regulation of MAP kinases by MAP kinase phosphatases. Biochim Biophys Acta BBA - Mol Cell Res. (2007) 1773:1227–37. doi: 10.1016/j.bbamcr.2006.12.002 17208316

[138] Yang Y Kim SC Yu T Yi Y-S Rhee MH Sung G-H. Functional roles of p38 mitogen-activated protein kinase in macrophage-mediated inflammatory responses. Mediators Inflammation. (2014) 2014:1–13. doi: 10.1155/2014/352371 PMC3977509 24771982

[139] Richardson ET Shukla S Nagy N Boom WH Beck RC Zhou L. ERK signaling is essential for macrophage development. PloS One. (2015) 10:e0140064. doi: 10.1371/journal.pone.0140064 26445168 PMC4596867

[140] Xu Z Lin R Hou X Wu J Zhao W Ma H. Immunomodulatory mechanism of a purified polysaccharide isolated from Isaria cicadae Miquel on RAW264.7 cells via activating TLR4-MAPK-NF-κB signaling pathway. Int J Biol Macromol. (2020) 164:4329–38. doi: 10.1016/j.ijbiomac.2020.09.035 32926903

[141] Liu C Cui Y Pi F Cheng Y Guo Y Qian H. Extraction, purification, structural characteristics, biological activities and pharmacological applications of acemannan, a polysaccharide from aloe vera: A review. Molecules. (2019) 24:1554. doi: 10.3390/molecules24081554 31010204 PMC6515206

[142] Li Q Verma IM. NF-κB regulation in the immune system. Nat Rev Immunol. (2002) 2:725–34. doi: 10.1038/nri910 12360211

[143] Hagemann T Lawrence T McNeish I Charles KA Kulbe H Thompson RG. Re-educating” tumor-associated macrophages by targeting NF-κB. J Exp Med. (2008) 205:1261–8. doi: 10.1084/jem.20080108 PMC2413024 18490490

[144] He Y Peng H Zhang H Liu Y Sun H. Structural characteristics and immunopotentiation activity of two polysaccharides from the petal of Crocus sativus. Int J Biol Macromol. (2021) 180:129–42. doi: 10.1016/j.ijbiomac.2021.03.006 33676979

[145] Wang D Wang J Liu H Liu M Yang Y Zhong S. The main structural unit elucidation and immunomodulatory activity *in vitro* of a selenium-enriched polysaccharide produced by pleurotus ostreatus. Molecules. (2022) 27:2591. doi: 10.3390/molecules27082591 35458788 PMC9027278

[146] Arora S Dev K Agarwal B Das P Syed MA. Macrophages: Their role, activation and polarization in pulmonary diseases. Immunobiology. (2018) 223:383–96. doi: 10.1016/j.imbio.2017.11.001 PMC7114886 29146235

[147] De Beule N De Veirman K Maes K De Bruyne E Menu E Breckpot K. Tumour-associated macrophage-mediated survival of myeloma cells through STAT3 activation. J Pathol. (2017) 241:534–46. doi: 10.1002/path.4860 27976373

[148] Yin H Zhang X Yang P Zhang X Peng Y Li D. RNA m6A methylation orchestrates cancer growth and metastasis via macrophage reprogramming. Nat Commun. (2021) 12:1394. doi: 10.1038/s41467-021-21514-8 33654093 PMC7925544

[149] Guo W Liu X Guo J Gao R Xiang X An X. Polysaccharides of *Brassica rapa* L. attenuate tumor growth via shifting macrophages to M1 -like phenotype. Phytother Res. (2022) 36:3957–68. doi: 10.1002/ptr.7545 35766285

[150] Kong F Chen T Li X Jia Y. The current application and future prospects of astragalus polysaccharide combined with cancer immunotherapy: A review. Front Pharmacol. (2021) 12:737674. doi: 10.3389/fphar.2021.737674 34721026 PMC8548714

[151] Du Y Wan H Huang P Yang J He Y. A critical review of Astragalus polysaccharides: From therapeutic mechanisms to pharmaceutics. BioMed Pharmacother. (2022) 147:112654. doi: 10.1016/j.biopha.2022.112654 35086031

[152] Cao D Xu H Xu X Guo T Ge W. A reliable and feasible way to predict the benefits of Nivolumab in patients with non-small cell lung cancer: a pooled analysis of 14 retrospective studies. Oncoimmunology. (2018) 7:e1507262. doi: 10.1080/2162402X.2018.1507262 30377569 PMC6205035

[153] Jiang T Bai Y Zhou F Li W Gao G Su C. Clinical value of neutrophil-to-lymphocyte ratio in patients with non-small-cell lung cancer treated with PD-1/PD-L1 inhibitors. Lung Cancer Amst Neth. (2019) 130:76–83. doi: 10.1016/j.lungcan.2019.02.009 30885355

[154] Guo L Bai S-P Zhao L Wang X-H. Astragalus polysaccharide injection integrated with vinorelbine and cisplatin for patients with advanced non-small cell lung cancer: effects on quality of life and survival. Med Oncol. (2012) 29:1656–62. doi: 10.1007/s12032-011-0068-9 21928106

[155] Davidson PJ Li S-Y Lohse AG Vandergaast R Verde E Pearson A. Transport of galectin-3 between the nucleus and cytoplasm. I. Conditions and signals for nuclear import. Glycobiology. (2006) 16:602–11. doi: 10.1093/glycob/cwj088 16473835

[156] Liu F-T Stowell SR. The role of galectins in immunity and infection. Nat Rev Immunol. (2023) 23:479–94. doi: 10.1038/s41577-022-00829-7 PMC9842223 36646848

[157] Fuselier C Dumoulin A Paré A Nehmé R Ajarrag S Granger Joly de Boissel P. Placental galectins in cancer: why we should pay more attention. Cells. (2023) 12:437. doi: 10.3390/cells12030437 36766779 PMC9914345

[158] MacKinnon AC Farnworth SL Hodkinson PS Henderson NC Atkinson KM Leffler H. Regulation of alternative macrophage activation by galectin-3. J Immunol. (2008) 180:2650–8. doi: 10.4049/jimmunol.180.4.2650 18250477

[159] Newlaczyl AU Yu L-G. Galectin-3–a jack-of-all-trades in cancer. Cancer Lett. (2011) 313:123–8. doi: 10.1016/j.canlet.2011.09.003 21974805

[160] Funasaka T Raz A Nangia-Makker P. Galectin-3 in angiogenesis and metastasis. Glycobiology. (2014) 24:886–91. doi: 10.1093/glycob/cwu086 PMC4153760 25138305

[161] Fang T Liu D-D Ning H-M Liu D Sun J-Y Huang X-J. Modified citrus pectin inhibited bladder tumor growth through downregulation of galectin-3. Acta Pharmacol Sin. (2018) 39:1885–93. doi: 10.1038/s41401-018-0004-z PMC6289393 29769742

[162] Miller MC Klyosov A Mayo KH. The alpha-galactomannan Davanat binds galectin-1 at a site different from the conventional galectin carbohydrate binding domain. Glycobiology. (2009) 19:1034–45. doi: 10.1093/glycob/cwp084 PMC2720280 19541770

[163] Chalasani N Abdelmalek MF Garcia-Tsao G Vuppalanchi R Alkhouri N Rinella M. Effects of belapectin, an inhibitor of galectin-3, in patients with nonalcoholic steatohepatitis with cirrhosis and portal hypertension. Gastroenterology. (2020) 158:1334–1345.e5. doi: 10.1053/j.gastro.2019.11.296 31812510

[164] Harrison SA Marri SR Chalasani N Kohli R Aronstein W Thompson GA. Randomised clinical study: GR-MD-02, a galectin-3 inhibitor, vs. placebo in patients having non-alcoholic steatohepatitis with advanced fibrosis. Aliment Pharmacol Ther. (2016) 44:1183–98. doi: 10.1111/apt.13816 27778367

[165] Ritchie S Neal D Shlevin H Allgood A Traber P. A phase 2a, open-label pilot study of the galectin-3 inhibitor GR-MD-02 for the treatment of moderate-to-severe plaque psoriasis. J Am Acad Dermatol. (2017) 77:753–5. doi: 10.1016/j.jaad.2017.05.055 28917454

[166] Redmond WL Gough MJ Weinberg AD. Ligation of the OX40 co-stimulatory receptor reverses self-Ag and tumor-induced CD8 T-cell anergy. vivo. Eur J Immunol. (2009) 39:2184–94. doi: 10.1002/eji.200939348 PMC3509800 19672905

[167] Sturgill ER Rolig AS Linch SN Mick C Kasiewicz MJ Sun Z. Galectin-3 inhibition with belapectin combined with anti-OX40 therapy reprograms the tumor microenvironment to favor anti-tumor immunity. Oncoimmunology. (2021) 10:1892265. doi: 10.1080/2162402X.2021.1892265 33717655 PMC7927986

[168] Bi S Huang W Chen S Huang C Li C Guo Z. Cordyceps militaris polysaccharide converts immunosuppressive macrophages into M1-like phenotype and activates T lymphocytes by inhibiting the PD-L1/PD-1 axis between TAMs and T lymphocytes. Int J Biol Macromol. (2020) 150:261–80. doi: 10.1016/j.ijbiomac.2020.02.050 32044366

[169] Rabinovich GA Liu F-T Hirashima M Anderson A. An emerging role for galectins in tuning the immune response: lessons from experimental models of inflammatory disease, autoimmunity and cancer. Scand J Immunol. (2007) 66:143–58. doi: 10.1111/j.1365-3083.2007.01986.x 17635792

